# Review and revamp of compositional data transformation: A new framework combining proportion conversion and contrast transformation

**DOI:** 10.1016/j.csbj.2024.11.003

**Published:** 2024-11-08

**Authors:** Yiqian Zhang, Jonas Schluter, Lijun Zhang, Xuan Cao, Robert R. Jenq, Hao Feng, Jonathan Haines, Liangliang Zhang

**Affiliations:** a Department of Population and Quantitative Health Sciences, Case Western Reserve University, 2109 Adelbert Rd, Cleveland, 44106, OH, USA; b Department of Statistics, University of Illinois Urbana-Champaign, 605 E. Springfield Ave., Champaign, 61820, IL, USA; c Institute for Systems Genetics, Department of Microbiology, New York University Grossman School of Medicine, 435 East 30th Street, New York, 10016, NY, USA; d Division of Statistics and Data Science, Department of Mathematical Sciences, University of Cincinnati, 2815 Commons Way, Cincinnati, 45219, OH, USA; e Department of Hematology & Hematopoietic Cell Transplantation, City of Hope, 1500 East Duarte Road, Duarte, 91010, CA, USA; f Case Comprehensive Cancer Center, 2103 Cornell Road, Cleveland, 44106, OH, USA

**Keywords:** Compositional data analysis, Contrast transformation, Conversion, Microbiome, Relative abundance, Zero inflation

## Abstract

Due to the development of next-generation sequencing technology and an increased appreciation of their role in modulating host immunity and their potential as therapeutic agents, the human microbiome has emerged as a key area of interest in various biological investigations of human health and disease. However, microbiome data present a number of statistical challenges not addressed by existing methods, such as the varying sequencing depth, the compositionality, and zero inflation. Solutions like scaling and transformation methods help to mitigate heterogeneity and release constraints, but often introduce biases and yield inconsistent results on the same data. To address these issues, we conduct a systematic review of compositional data transformation, with a particular focus on the connection and distinction of existing techniques. Additionally, we create a new framework that enables the development of new transformations by combining proportion conversion with contrast transformations. This framework includes well-known methods such as Additive Log Ratio (ALR) and Centered Log Ratio (CLR) as special cases. Using this framework, we develop two novel transformations—Centered Arcsine Contrast (CAC) and Additive Arcsine Contrast (AAC)—which show enhanced performance in scenarios with high zero-inflation. Moreover, our findings suggest that ALR and CLR transformations are more effective when zero values are less prevalent. This comprehensive review and the innovative framework provide microbiome researchers with a significant direction to enhance data transformation procedures and improve analytical outcomes.

## Graphical abstract

**Figure:**
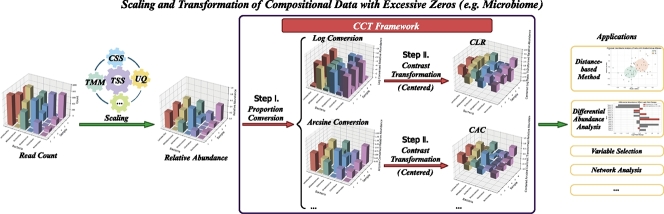

## 1 Introduction

The vast family of microorganisms, including bacteria, fungi, and viruses, outnumbers human cells by approximately ten to one, and is integral to human physiology, affecting various bodily functions and maintaining homeostasis. Unique microorganisms inhabit different sites on the body, each adapted to the specific environment and function needs of its location. Eating certain foods, like farmed animal meat, dairy products, refined vegetable oils, and processed cereals, changes the oral microbiota composition, increasing acid-producing, acid-tolerant organisms, and periodontal pathogens [1]. The gut microbiome, for instance, is essential for breaking down complex carbohydrates, synthesizing vitamins, and modulating immune responses [2], [3]. The significance of these microbes has been further highlighted by the Human Microbiome Project, which demonstrates their contributing role in metabolic functions that extend beyond the scope of human genetics alone [3], [4], [5].

Research on human microbiome is revolutionizing our understanding of its pivotal role in sustaining health and influencing the progression of diseases such as cancer, cardiovascular diseases, allergies, and obesity [6]. When the balance of microbiota is disrupted, a condition known as dysbiosis, can lead to various health issues. For example, changes in gut microbiota composition are associated with diseases such as colorectal cancer, where microbial metabolites can influence carcinogenesis [7]. Similarly, cardiovascular diseases have been linked to microbial metabolites like Trimethylamine N-oxide (TMAO), which can contribute to atherosclerosis [8]. Furthermore, the microbiota plays a crucial role in immune system development and regulation, with early-life microbiota influencing long-term immune health [2]. A cancer research study reveals that higher alpha-diversity of the tumor microbiota in long-term pancreatic adenocarcinoma survivors is linked to improved survival [9].

Despite these critical roles, analyzing microbiome data presents several statistical challenges due to the complexities introduced by high-throughput sequencing (HTS) techniques used to generate these datasets. First, differences in sequencing depth across samples make comparisons challenging. Variations in the number of sequences representing the microbial community often result from differences in sequencing efficiency rather than true biological variations. Additionally, because the full diversity of bacterial species is rarely captured, more species are discovered as sequencing efforts are increased [10], [11], [12], [13]. Second, microbiome read counts, obtained through 16S rRNA marker gene sequencing or metagenomic shotgun sequencing, often exhibit high sparsity, with as many as 95% zeros. This high level of sparsity introduces uncertainty in the detection and quantification of rare taxa [13], [14], [15]. Moreover, existing methods struggle to distinguish between different types of zeros, which are categorized as biological zeros (when a taxon is truly absent), sampling zeros (due to sequencing depth limitations), and technical zeros (resulting from sample preparation errors) [16]. Third, HTS datasets inherently provide only relative abundances of microbial populations, constrained by the sequencing instrument's capacity, rather than absolute counts [13], [17]. Adding sequences from one taxon reduces sequences from another, causing misinterpretations if the compositional nature is ignored. Using compositional data analysis methods such as log-ratio transformations is crucial to avoid spurious correlations and gain accurate insights into microbial communities [18].

Preprocessing microbiome data through scaling and transformation is critical to prepare it for downstream analyses, helping to reduce biases and recover true biological signals. Scaling involves dividing read counts by a scale factor to adjust for discrepancies in sequencing depth and other technical variations, ensuring comparability across samples [19]. Total sum scaling (TSS) is a specific scaling method that divides read counts by the total count in each sample [14], [20], producing relative abundances that are both proportional and compositional. Transformation of relative abundance data involves removing the constant sum constraint [21]. This constraint introduces interdependence between variables, which can mislead statistical analyses if traditional multivariate methods are used without adjustment [22], [23].

Over the past decade, various scaling and transformation techniques, along with differential abundance (DA) analyses have been developed to identify key microbial taxa in host-health-microbiome association studies. However, analyses of the same microbiome data often yield divergent findings, highlighting the lack of consensus and resulting in heterogeneous conclusions [24], [25], [26]. In addition, debates between count data analysis [27], [28], [29], [30] and compositional data analysis (CoDA) [31], [32], [33], [34] in the context of microbiome research are ongoing and touch upon several key methodological and theoretical aspects. The field still faces significant gaps, including a lack of comprehensive statistical validation and consistent framework to produce robust results.

**Fig. 1:**
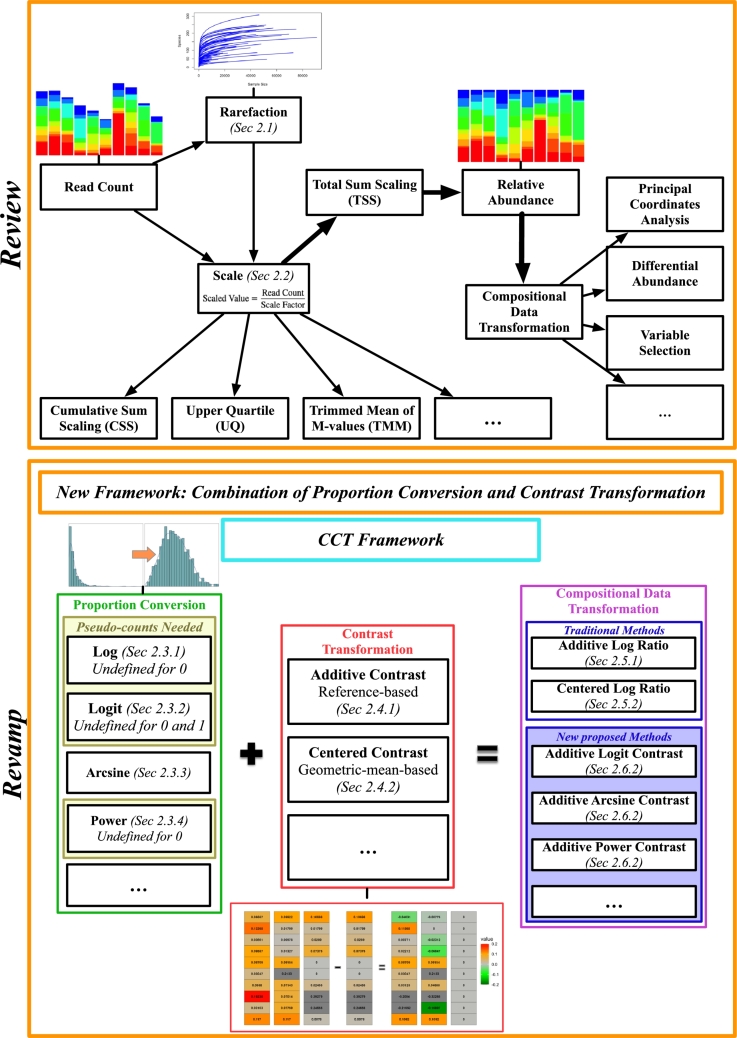
An integrated framework for microbiome data transformation. This framework addresses critical challenges in microbiome data analysis by combining proportional conversion with contrast transformation techniques. The goal is to achieve normal or quasi-normal distributions, facilitating robust statistical analysis and ensuring the reliability and validity of the results.

To address these questions, we will conduct a systematic review of count data scaling and compositional data transformation, with a particular focus on the connection and distinction of existing techniques. Our goal is to unify and refine compositional data transformation approaches, developing new methods to manage within-sample compositionality and across-sample variability. We will create a framework for proposing novel compositional transformations by combining proportion conversion and contrast transformation. As shown in Fig. 1, proportion conversion stabilizes variance and reduces the influence of outliers, while contrast transformation handles compositionality. The framework includes Additive Log Ratio (ALR) and Centered Log Ratio (CLR) as special cases, while enriching the range of potential options. We will study the statistical properties of different combinations in terms of variance stabilization, handling zero values, and sensitivity to outliers. These novel transformations strive to achieve a normal or quasi-normal distribution of the transformed data, allowing the use of basic statistical tests, such as the t-test, to assess their effectiveness. This innovative approach provides microbiome researchers with a significant direction to enhance data transformation procedures and improve analytical outcomes.

## 2 Methods

Microbial sequence abundance has intrinsic data characteristics that prevent accurate recovery of the population composition within its original environment. Different samples often yield different total read counts due to variations in sequencing depth. To mitigate sequencing depth variability, researchers often adopt rarefaction methods [35], originally proposed in ecology. These methods involve subsampling to a uniform depth to control the effects of uneven sequencing. Alternatively, scaling preserves all the data and is employed to ensure that inherent differences do not bias results, thereby facilitating accurate comparisons across samples. However, the effectiveness of scaling methods can vary depending on the context, leading to differing interpretations of community structure and composition, which may limit the generalizability of results derived from the same dataset. Total sum scaling (TSS) [36] is a popular scaling method that preserves relative abundance information, making it suitable for comparing the microbial community composition across samples. Relative abundances are both proportional and compositional in nature [13], [37]. Therefore, there are two aspects to consider when transforming compositional data. First, converting proportional data enhances the symmetry of the distribution, stabilizes variance, and controls the effects of outliers. Second, contrast transformation constructs relative changes between compositions, facilitating unconstrained analysis in Euclidean space. In the following section, we provide an in-depth review of these methods, highlighting their specific advantages and disadvantages. We analyze various proportion conversion techniques, contrast transformations, and their practical implications. Additionally, we explore potential alternative solutions that could address existing limitations and improve the accuracy and reliability of microbiome data analysis.

### 2.1 Rarefaction of read counts

Rarefaction was first developed by Howard Sanders in 1968 to compare species richness data among sets with different sample sizes in marine ecology research [35]. The primary motivation behind its development was to create a method that would allow fair comparisons between datasets with unequal sampling efforts. This method is essential for assessing the diversity of sequencing data, as it standardizes sampling depth, allowing for accurate comparisons of diversity between environments. Without rarefaction, deeper sequencing can artificially inflate diversity by detecting rare taxa that might be missed in shallower-sequenced samples, leading to biased alpha (within-sample) and beta (between-sample) diversity estimates [13].

Rarefaction works by selecting a fixed number of samples, equal to or less than the smallest sample in the dataset, and randomly subsampling the larger datasets by discarding reads until the sample sizes match this threshold [38]. This subsampling to a common depth also maintains the exchangeability of observations under the null hypothesis, thereby controlling the Type I error rate in permutation-based statistical tests [39]. Rarefaction curves are valuable for assessing both sample coverage and the adequacy of threshold for reliable diversity estimates [40]. Moreover, rarefaction is straightforward to implement and widely supported in various bioinformatics tools, making it accessible for researchers [40], [41].

When determining rarefaction depth, researchers must balance sampling breadth and sequencing depth. Greater breadth increases statistical power for comparing treatment groups, while greater depth improves the resolution of microbial community characterization [42]. Lower sequencing depth, however, may result in significant data loss through rarefaction, reducing statistical power and increasing variance, which decreases the sensitivity of analyses and makes it harder to detect true differences in microbial composition [25], [39], [43]. While McMurdie and Holmes [43] argued that rarefaction could increase false positives and reduce analysis sensitivity due to data reduction and added variability [25], [39], more recent studies support its continued use in microbiome research. Schloss [42], [44] countered these claims, emphasizing that rarefaction remains the most reliable method for controlling sequencing depth variation in both alpha and beta diversity analyses. Their simulations show that rarefaction preserves statistical power and limits false positives, particularly when sequencing effort is confounded with treatment groups.

In the context of differential abundance analysis, rarefaction might not be necessary unless there's a strong correlation between sequencing depth and the variables of interest. Instead, scaling techniques are generally preferred for differential abundance analysis because they retain the full data set and provide more reliable results in the context of compositional data.

### 2.2 Scaling of count data

Scaling is a straightforward and commonly used statistical method that corrects observed counts by dividing them by sample-specific scale factors, aiming to mitigate discrepancies in sequencing depth [45]. The rationale behind scaling lies in its ability to correct for technical variability while preserving the biological integrity of the data. By normalizing counts using total reads or other summary statistics, scaling ensures that comparisons between samples reflect true biological variation rather than artifacts from uneven sequencing.

Before delving into specific scaling methods, we suggest categorizing scaling methods into two main types to enhance clarity. This classification structure is shown in Fig. 1. The first type of scaling, which we refer to as Depth-Adjusted Abundance, retains the data in a count-like format after scaling, such as Cumulative Sum Scaling (CSS) [14], Upper Quartile (UQ) [20], Trimmed Mean of M-values (TMM) [46], Counts Per Million (CPM) [29], [30], and Geometric Mean of Pairwise Ratios (GMPR) [47]. The second type, which we refer to as Relative Abundance, transforms the data into proportions where the sum of all taxa within each sample equals one [27]. This scaling provides a true compositional view of the data. Total Sum Scaling (TSS) [36] is a common method in this category, as it directly scales count data into relative abundances. Given the complexity and variability inherent in microbiome data, choosing the appropriate scaling method is crucial for ensuring accurate and reliable analysis. We will begin by examining the first type of scaling.

**Cumulative Sum Scaling (CSS)**, which is used in metagenomeSeq [14], assumes that observed abundances are roughly independent and identically distributed up to a specific quantile [14]. This method was originally proposed to better separate samples based on biological factors while controlling within-group variance [14]. The motivation behind CSS was to create a scaling technique that minimizes the influence of highly abundant taxa, which can skew results in datasets with a wide range of microbial abundances. Traditional scaling techniques, such as TSS, are heavily influenced by a few highly abundant taxa, leading to biased estimates of relative abundance. By focusing on the cumulative sum up to a certain quantile, CSS provides a more stable and representative scaling factor that is less sensitive to extreme values [27], [45], [48]. However, determining the optimal quantile can be challenging due to high count variability, potentially affecting the scaling process [14], [45].

**Upper Quartile (UQ)** Scaling uses the upper quartile of observed abundances as the scaling factor, aiming to capture the invariant segment of the count distribution [20], [49], [50]. Like CSS, the motivation behind UQ Scaling is to develop a scaling method that minimizes the influence of highly abundant taxa, which can skew the scaling factor in traditional methods like Total Sum Scaling (TSS). By focusing on the upper quartile, UQ Scaling ensures that the scaling process remains stable even in the presence of extreme values. UQ Scaling is robust as it reduces the impact of extremely high counts from a few taxa. However, as CSS, selecting the most effective quantile remains nontrivial and can influence the scaling's effectiveness [27]. This challenge is particularly evident in datasets with high count variability, where a suboptimal choice of quantile can lead to under- or over-adjustment of abundances. Additionally, the study by Pereira et al. [51] indicates that for shotgun metagenomic data, TSS method has been evaluated and shown to perform on par with or surpass the UQ method.

**Trimmed Mean of M-values (TMM)** scaling adjusts for library sizes by selecting a reference sample, typically with a median library size, and calculating log-fold changes (M-values) between this reference and each other sample for each gene. The motivation behind TMM scaling is to provide a robust method that accounts for compositional differences between samples, especially in datasets with varying library sizes and potential biases introduced by highly expressed genes. TMM assumes that most OTUs (ASVs/genes) are not differentially abundant, and that overall abundances between samples should be similar on average. The process involves filtering OTUs based on their mean abundance and fold-change relative to the reference, effectively trimming extreme M-values to avoid outliers. This trimming helps to reduce the impact of highly expressed genes and extreme values, leading to more reliable scaling. A weighted mean of the remaining log-fold changes is then calculated, where weights are the inverse of the variance [27], [46], [50]. However, the assumptions underlying TMM scaling, such as the belief that most OTUs are not differentially abundant, may not be suitable for highly diverse microbial environments [13].

**Counts Per Million (CPM)** scaling, or called Reads Per Million (RPM) scaling, is a simpler scaling technique where raw counts are scaled by the total number of reads in each sample, then multiplied by one million. This method adjusts for sequencing depth differences by expressing counts on a per-million-reads basis, allowing straightforward comparisons across samples [29], [30]. However, CPM does not account for compositional biases, which can be significant in microbiome data.

**Geometric Mean of Pairwise Ratios (GMPR)** scaling, builds on the concept of Relative Log Expression (RLE) used for RNA-seq data [47], [52], provides a robust alternative by using the geometric mean of pairwise ratios of counts between samples to calculate scaling factors. GMPR is particularly effective for microbiome data as it accounts for compositional differences and handles zeros and varying sequencing depths robustly [47], [53]. By focusing on pairwise comparisons, GMPR reduces the impact of outliers and rare taxa, resulting in more reliable normalization across diverse microbial communities. This method enhances traditional approaches by using the median count ratio of nonzero counts between samples to calculate the geometric mean for size factors, and it is based on the moderated estimation of dispersion (MED) in the DESeq2 method [28].

The second type of scaling, **Total Sum Scaling (TSS)**, proposed by Bergemann and Wilson [36] in RNA-seq data, is a method that scales individual read counts by the total number of reads. This process transforms absolute abundances into relative abundances, which are compositional and sum to 1. According to McKnight et al. [54], TSS outperformed other scaling methods in producing accurate Bray-Curtis dissimilarities [55], [56], principal coordinates analysis, and PERMANOVAs, avoiding spurious correlations [52]. This makes TSS highly effective for community-level comparisons in microbiome studies. Many biological interpretations and downstream analyses, such as diversity indices and ecological modeling, are based on these proportions rather than absolute counts [57]. By focusing on the proportionate presence of taxa, TSS mitigates biases introduced by overdispersion or sequencing errors. Additionally, TSS adjusts for differences in sequencing efforts and efficiencies between samples, providing a more accurate reflection of the microbial community structure [52]. However, TSS has limitations, including potential biases in differential abundance estimates and a high rate of false positives due to the influence of highly abundant taxa [13], [45], [47], [50].

In summary, while extensive discussion has focused on count data in microbiome research, there has been limited review and systematic evaluation of relative abundance transformations, such as TSS. TSS actually connects count data scaling with compositional data transformation. TSS scales each count by the total number of reads in the sample, effectively reconstructing count data into relative abundance, which ensures comparability across samples and studies and reducing biases. To analyze relative abundance effectively, compositional data approaches are required to transform data on the simplex to Euclidean space. In the following paper, we will focus on TSS and relative abundance. Since microbial relative abundance is both proportional and compositional, our review will be structured into two parts: conversion of proportions and transformation of compositions.

### 2.3 Conversion of proportional data

In microbiome research, each column of a relative abundance table represents a proportional variable. Proportional data, expressed as percentages or fractions of a whole, are scale-independent and commonly analyzed across various biological subfields, making them suitable for studying many biological phenomena. Proportional data can be formally understood as the division of a total *W* (e.g., counts, area, time, mass) into *C* parts or categories [58]. Statistical analysis of proportional data presents numerous challenges due to their bounded nature between 0 and 1. The variability in the observed proportions usually varies systematically with the mean of response [58]. To address these issues, mathematical functions such as logarithm or logit are often applied to the proportional data—a process we refer to as “conversion”. However, applying these conversions can lead to biased estimates and interpretation difficulties [58]. We chose the term “conversion” instead of “transformation” to avoid confusion with contrast transformations, which we will discuss later in our manuscript.

We begin by exploring several common conversion methods for proportional data, including log, logit, arcsine, and power conversion. Each conversion is detailed with its mathematical formula, along with its advantages and disadvantages. Table 1 provides a detailed summary of the distributions used for traditional proportional data conversion. After detailing the conversion, we simulate proportional data using zero-inflated beta regression, which is well-suited for modeling proportions, to evaluate the power of each conversion method. Additionally, we employ simple linear regression to generate data with varying levels of variance and outliers and evaluate the efficiency of these traditional conversion (log, logit, arcsine, Box-Cox) in reducing variance and managing outliers. By comparing these conversions, we aim to identify the most effective methods for stabilizing variance in proportional data and improving its interpretability, which will serve as a basis for more complex compositional data transformation.

**Table 1:** Distributions and corresponding conversion methods, formulas, and intervals.

Conversion Method	Formula	Interval	Corresponding Distribution	Distribution in Mathematical Formula	Reference
Log Conversion	y=log⁡(x)	*x* > 0	Log Normal Distribution	$f(x;\mu ,\sigma )=\frac{1}{x\sigma \sqrt{2\pi }}\exp\left(-\frac{{\left(\ln x-\mu \right)}^{2}}{2\sigma^{2}}\right)$	Crow and Shimizu [59]
Logit Conversion	$y=\log\left(\frac{x}{1-x}\right)$	0 < *x* < 1	Logit Normal Distribution	$f(x;\mu ,\sigma )=\frac{1}{x(1-x)\sigma \sqrt{2\pi }}\exp\left(-\frac{{\left(\log\left(\frac{x}{1-x}\right)-\mu \right)}^{2}}{2\sigma^{2}}\right)$	Atchison and Shen [60]
Arcsine Conversion	$y=\frac{2}{\pi }\arcsin(\sqrt{x})$	0 ≤ *x* ≤ 1	Arcsine Normal Distribution	$f(x;\mu ,\sigma )=\frac{1}{\sigma \sqrt{2\pi }}\exp\left(-\frac{{\left(\frac{2}{\pi }\arcsin\left(\sqrt{x}\right)-\mu \right)}^{2}}{2\sigma^{2}}\right)\cdot \frac{1}{\pi \sqrt{x}\sqrt{1-x}}$	Proposed
Box-Cox Conversion	$y=\left\{ \begin{array}{cccc} & \frac{x^{\lambda }-1}{\lambda } & \; & \text{for }\lambda \neq 0\\ & \log(x) & \; & \text{for }\lambda =0 \end{array}\right.$	*x* > 0	Power Normal Distribution	$f(x;\mu ,\sigma ,\lambda )=\frac{x^{\lambda -1}}{\sigma A(\lambda ,\mu ,\sigma )}\phi \left(\frac{x^{\left(\lambda \right)}-\mu }{\sigma }\right)$	Gonçalves [61]

From 2021 to 2024, the popularity and usage trends of different conversion methods in microbiome research were examined through Google Scholar searches. In 2021, Log conversion was the most widely used, with 17,400 results, followed by Logit conversion with 6,150 results, and Arcsine and Box-Cox conversions with 641 and 464 results, respectively. The trend continued in 2022, with Log conversion reaching a peak of 24,700 results, Logit conversion increasing to 7,030 results, Arcsine conversion rising to 723 results, and Box-Cox conversion going up to 496 results. In 2023, usage slightly declined, with Log conversion at 17,000 results, Logit conversion at 5,420 results, Arcsine conversion at 548 results, and Box-Cox conversion at 378 results. By 2024, Log conversion decreased to 4,480 results, Logit conversion dropped to 1,820 results, Arcsine conversion had 181 results, and Box-Cox conversion had 122 results.

Overall, Log conversion is the most widely used method, followed by Logit conversion, while Arcsine and Box-Cox conversions are less common, with Box-Cox being the least used. These trends suggest that researchers in microbiome studies favor certain conversion methods, possibly because they effectively fit the nature of the data. The peak usage of most conversion methods in 2022 may indicate particularly high research activity or publications in that year.

#### 2.3.1 Log conversion

The history of logarithms dates back to John Napier's invention in 1614, as detailed in his work “Mirifici Logarithmorum Canonis Descriptio,” which represents one of the greatest scientific discoveries, providing a significant advancement in mathematical science and a labor-saving tool for extensive numerical calculations [62].

The log conversion transforms multiplicative relationships into additive ones, thereby simplifying the analysis of multiplicative models. Following the conversion, exponential growth patterns may appear linear, facilitating the implementation of simpler linear modeling techniques. This is particularly advantageous when dealing with data where the variance is proportional to the square of the mean or where the effects are multiplicative, conditions commonly found in biological data such as growth measurements or insect counts [63].

Mathematical form of log conversion, defined as y=log⁡(x), assuming *x* represents the proportional data, is commonly used to shape right skewed data by making the distribution more symmetric. However, if the data is left-skewed, log conversion will worsen the left skew, moving it further away from a normal distribution.

It is important to note that when *x* ranges from 0 to 1, the log conversion log⁡(x) ranges from −∞ to 0. This means that the log conversion cannot handle zero values because log⁡(0) is undefined (it tends towards negative infinity). Therefore, a small positive constant is often used to replace 0 in *x* before applying the log conversion to avoid this issue. The selection of this small constant is crucial, as even minor variations can lead to significant differences in the transformed data. For example, $\log(10^{-5})=-5$ and $\log(10^{-2})=-2$. This issue is particularly pertinent in microbiome data, where a high proportion of zeros is common. Selecting a replacement value that, after conversion, becomes a small negative number far removed from other data values can lead to potential issues in data conversion. As highlighted by Changyong et al. [64], the p-value of the test can depend on the value added before applying the log conversion, potentially making conclusions about differences between groups reliant on the arbitrary decision regarding the size of the constant used in the analysis.

People believe that the log conversion can reduce variance and the impact of outliers [63]. However, for proportional data, things are different. Contrary to popular belief, the log conversion can sometimes increase the variability of data, whether or not there are outliers [64]. This is particularly true for data with a small mean, such as proportional data. Changyong et al. [64] recommend caution when applying log conversion and emphasize that researchers must be mindful about its limitations when using this method.

Despite its many shortcomings, the log conversion is a foundational method in microbiome data analysis, underpinning many commonly used techniques such as ALR and CLR [22]. These methods help in dealing with compositional data and making it suitable for various statistical analyses.

#### 2.3.2 Logit conversion

While the log conversion can merely handle right-skewed data, logit conversion is capable of managing both left-skewed and right-skewed data. The logit conversion is defined as the natural logarithm of the odds of an event occurring, expressed as $y=\log\left(\frac{x}{1-x}\right)$, where *x* is the proportion of interest and must lie within the domain 0<x<1. The logit conversion has its roots in the work of Pierre-François Verhulst [65], who first introduced the logistic function in 1838 to describe population growth. Verhulst's work remained largely unnoticed until the early 20th century when Raymond Pearl and Lowell Reed revived interest in the logistic function, fitting it to U.S. Census data to model population dynamics [66]. However, the development of logit conversion as we know it today owes much to Joseph Berkson [67]. In 1944, Berkson proposed using the logistic function in bio-assay and coined the term “logit” [67]. He advocated for the logit model as a simpler and more computationally efficient alternative to the probit model, which was prevalent at the time [68], [69]. The primary purpose of the logit model is to facilitate the analysis of binary outcomes, such as survival versus death or success versus failure, by transforming probabilities into log-odds. This conversion is essential in logistic regression, enabling the modeling of relationships between a binary dependent variable and multiple independent variables.

However, it is crucial to recognize that the logit conversion has limitations at the boundaries of the proportion scale. Specifically, it cannot directly handle proportions of exactly 0 or 1. Berkson [67] addresses this limitation by noting that for proportions, such as observed mortalities at zero or 100 percent, logit conversion becomes infinite. This limitation arises due to the mathematical implications of division by zero and taking the logarithm of zero in these cases. Consequently, in practical applications, the values of *x* are typically assumed to be within the open interval (0,1) to avoid these undefined operations.

Both log and logit conversion share the common objective of reconstructing skewed data into a more symmetric distribution, facilitating subsequent statistical analyses [70]. They are particularly useful in handling data with wide ranges and mitigating the impact of values close to 0 and 1 [70]. Despite their differences in handling data at the boundaries, both transformations convert multiplicative relationships into additive ones, aiding in linear regression and other parametric analyses [71]. Moreover, both methods are grounded in the principle of converting proportions and probabilities to a scale that enhances the interpretability and robustness of the data [70].

#### 2.3.3 Arcsine conversion

Log and logit conversions can transform proportional data but struggle at the boundaries of 0 or 1. To circumvent this, small value replacements are often used, however, may introduce biases and reduce the robustness of the analysis. Alternatively, the arcsine conversion is well defined on boundaries and presents a viable solution. The arcsine conversion, proposed by Sokal and Rohlf [72], is defined as $y=\frac{2}{\pi }\arcsin(\sqrt{x})$. It has been widely used in the analysis of proportional data due to its ability to stabilize variances. This conversion converts proportions, which are bounded between 0 and 1, into values between 0 and 1. One of the key advantages of the arcsine conversion is its ability to handle boundary values of 0 and 1, making it particularly useful for datasets that include lots of such boundary values. Specifically, $\frac{2}{\pi }\arcsin(\sqrt{0})=0$ and $\frac{2}{\pi }\arcsin(\sqrt{1})=1$. This ensures that the conversion is applicable across the entire range of proportion data, providing a robust method for statistical analysis [73].

However, the arcsine conversion has been criticized for its lack of interpretability and the fact that it can produce nonsensical predictions [74]. The criticism mainly stems from the fact that while the arcsine conversion stabilizes variances, it does not necessarily normalize the data well, and its predictions can be difficult to interpret in a meaningful way. One key issue is that the arcsine conversion maps 0 to 0. So when there is a high proportion of 0 in the data, the zeros remain unchanged after the conversion. This results in fewer nonzero values, limiting the transformed data's ability to approximate a normal distribution. However, zero-inflation poses similar challenges for all conversions.

**Fig. 2:**
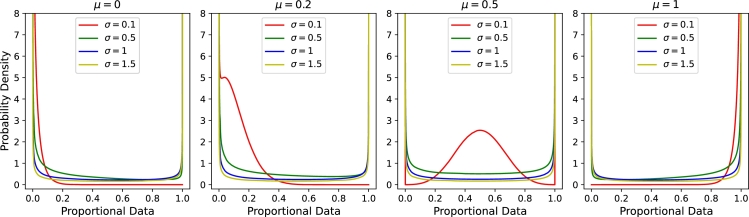
Probability density functions (PDFs) of the arcsine normal distribution for various parameter settings, illustrating their behavior and suitability for different types of proportion data.

To facilitate the normality of transformed data, we propose and derive the arcsine normal distribution as a new method to transform and analyze proportion data. As noted in Table 1, the arcsine conversion results in a normal distribution when applied to data following an arcsine normal distribution. To illustrate the characteristics of the arcsine normal distribution, we plot the probability density functions (PDFs) of the arcsine normal distribution under various parameter settings. Fig. 2 shows these distributions, highlighting their flexibility and suitability for different types of proportion data. This visualization demonstrates that the arcsine normal distribution can provide a valuable tool for analyzing proportion data, particularly when dealing with boundary values. For the full derivation of the arcsine normal distribution, refer to Supplementary Section 1.

As we can summarize from Table 1 and Fig. 2, as *x* approaches 0, the density of the arcsine normal distribution can tend to infinity. Because arcsine function is well defined at 0 and converts 0 to 0 (with a probability of 1). This behavior is primarily influenced by the Beta kernel $\frac{1}{\pi \sqrt{x}\sqrt{1-x}}$, which always tends to infinity as *x* approaches 0. However, the overall density is moderated by the Gaussian kernel $\exp\left(-\frac{{\left(\frac{2}{\pi }\arcsin\left(\sqrt{x}\right)-\mu \right)}^{2}}{2\sigma^{2}}\right)$. When *μ* is low and *σ* is high, this term does not significantly reduce the density, leading to a sharp increase near 0. Conversely, when *μ* is high or *σ* is low, the exponential term becomes very small, causing the density to approach extremely large values near 0—values that are too small to observe effectively. Similarly, as *x* approaches 1, the density can also go to infinity, but if *μ* is small and *σ* is small, the exponential part can moderate it, resulting in the density approaching extremely large values very close to 1. This behavior is in stark contrast to the log-normal and logit-normal distributions, where the density approaches 0 as *x* approaches 0. This indicates that when a dataset has a high percentage of zero values, or is highly skewed to the left (with most data points being extremely small), the arcsine normal distribution may perform better in representing the data's distribution. This property makes the arcsine conversion particularly suitable for analyzing datasets with these characteristics.

#### 2.3.4 Power conversion

The power conversion, often referred to as the Box-Cox conversion [75], is widely used in various fields, including economics, engineering, and the natural sciences, due to its flexibility and ability to handle different types of data distributions. Its application has been shown to improve the performance of statistical models by making the data more closely conform to the assumptions of normality and homoscedasticity [76], [75], [77]. It is defined as follows:

$$y=\left\{ \begin{array}{cc} \frac{x^{\lambda }-1}{\lambda } & \text{if }\lambda \neq 0\\ \log(x) & \text{if }\lambda =0 \end{array}\right.$$

where *y* is the transformed variable, *x* is the original variable (which must be positive), and *λ* is the conversion parameter. The power conversion can take various forms depending on the value of *λ*. When λ=0, the conversion is equivalent to a logarithmic conversion. When λ=1, it becomes an identity conversion, meaning no conversion is applied. Other values of *λ* result in different power conversions of the original variable. The choice of *λ* is critical and is typically selected to maximize the normality of the transformed data. This selection is often done empirically or through optimization techniques.

The power conversion is especially useful in transforming non-normal data into a normal distribution, which is a common prerequisite for many statistical methods such as regression analysis, analysis of variance, and t-tests [76], [78]. The transformation helps in stabilizing variance and making the data more symmetric, which enhances the validity of statistical inferences [77].

However, the power conversion has limitations, particularly when dealing with values of *x* that include zero. Since when λ=0, log⁡(0) is undefined and the conversion requires *x* to be positive, a common approach is to use a small positive constant to replace zero in all values of *x* before applying the conversion to avoid this issue. These adjustments ensure that the conversion can be applied to datasets that include zero, although they may introduce some bias [76]. The small constant added or used to replace zeros may also influence the *λ* for Box-Cox; for the same dataset, choosing different constants may result in different *λ* values.

#### 2.3.5 Other traditional conversion

In addition to the commonly used conversion techniques, there are several other transformations frequently employed for normalizing and analyzing proportion data. These include the Anscombe, probit, inverse hyperbolic sine, and tangent transformations. We provide a brief introduction to these methods without delving into detailed explanations.

The Anscombe conversion [79], expressed as $y=2\sqrt{x+\frac{3}{8}}$, plays a pivotal role in statistical analysis, especially in scenarios involving binomially distributed data. This transformation is frequently utilized in linear regression and Analysis of Variance (ANOVA) to satisfy key assumptions such as homogeneity of variance and normality of residuals. A notable feature of the Anscombe transformation is its inclusion of the adjustment term $\frac{3}{8}$, which ensures appropriate behavior across the entire [0,1] interval, including boundary values [80].

The probit conversion [68], defined as $y=\Phi^{-1}(x)$, where Φ(x) represents the cumulative distribution function (CDF) of the standard normal distribution, is another key statistical transformation. This conversion transforms a variable *x*, which follows a uniform distribution between 0 and 1, into a variable *y* that follows a standard normal distribution. However, the probit transformation is undefined at the boundary values of x=0 and x=1, which can pose challenges in practical applications.

The inverse hyperbolic sine (IHS) conversion, defined as $y=\log\left(x+\sqrt{1+x^{2}}\right)$, can handle zero and negative values. For most values of *y*, it is approximately equal to log⁡(2x), making it interpretable similarly to a standard logarithmic variable. Unlike the log conversion, the IHS is defined at zero, making it a robust alternative for certain datasets [81], [82].

The tangent conversion, expressed as y=tan⁡(π(x−0.5)), is another transformation used in statistical analysis. Suppose *x* follows a uniform distribution from 0 to 1, then y=tan⁡(π(x−0.5)) will follow a Cauchy distribution. The Cauchy distribution does not have a mean or variance, which precludes the use of traditional statistical methods such as the two-sample t-test. However, the Cauchy combination test can be applied [83], [84].

#### 2.3.6 Proposed new conversion for proportions

Microbiome data, which often exhibit zero inflation, present significant challenges for traditional conversion methods like log and logit. These methods typically cannot handle zero values, as they are undefined for zero. Using small constants to replace zeros introduces bias and distorts data distribution, as there is no mathematical justification for the chosen constant's magnitude. To address this issue, we propose a truncated Logit conversion with adjustable parameters, defined as $y=\log\left(\frac{x+\phi }{1-x+\varphi }\right)$. Here, ϕ>0 and φ>0 ensure that logit conversion remains well-defined even when x=0 or x=1, thus accommodating the zero-inflation often observed in microbiome data. This introduction of adjustable parameters is inspired by the Box-Cox transformation [77], [78], where the power parameter *λ* is varied to adjust data distribution characteristics. We expect that the transformed data will approximate a normal distribution, denoted as $N(\mu ,\sigma^{2})$. Given the four parameters in the joint likelihood function, we optimize and estimate the adjustable parameters through profile likelihood maximization. This new conversion enhances flexibility by incorporating adjustable parameters, refining small constant selection to ensure a well-defined and robust conversion.

In studies comparing two groups to identify differential features, we introduce and clarify the concept of dual group conversion method. Instead of assuming a single normal distribution, we assume the transformed data will approximate two distinct normal distributions: $N(\mu_{A},\sigma_{A}^{2})$ for group A and $N(\mu_{B},\sigma_{B}^{2})$ for group B, while using same adjustable parameters across both groups. More specifically, given two groups *A* and *B*, we apply the truncated logit conversion to data from two groups $x^{A}$ and $x^{B}$ as follows:

$$y^{A}=\log\left(\frac{x^{A}+\phi }{1-x^{A}+\varphi }\right),\;y^{B}=\log\left(\frac{x^{B}+\phi }{1-x^{B}+\varphi }\right),$$

where ϕ>0,φ>0 are the shared truncation parameters at 0 and 1 respectively. Given the six parameters in the joint likelihood function, we estimate the adjustable parameters through profile likelihood maximization. Since our approach aims to approximate distinct normal distributions across two groups, we have named our method as the **Dual-Group Truncated Logit conversion (DGTL)**. For a detailed derivation of DGTL, please refer to Supplementary Section 3.2. This dual-group conversion structure offers a valuable and streamlined approach for differential abundance analysis in microbiome studies, yet it has rarely been systematically explored or clearly defined.

This technique can also be applied to other conversions, such as the Box-Cox conversion (detailed in the Supplementary Section 3.3 as the **Dual-Group Box-Cox Conversion (DGBC)**). By incorporating dual group considerations and maintaining the same adjustable parameters, this framework achieves an effective balance between preserving pre-conversion information and enhancing the power to detect differential abundance.

#### 2.3.7 Comparison of conversions

We compare the effectiveness of various conversion methods using simulated data from a zero-inflated beta regression model. The performance of each transformation is evaluated based on power, false discovery rate (FDR), and standard deviation (SD) when applied to two-group comparisons using the two-sample t-test and the Wilcoxon rank sum test [85]. This approach assesses both parametric and non-parametric methods in handling transformed data and their ability to detect significant differences between groups.

Data simulation is conducted using a zero-inflated beta regression model, with significant variables influenced by the covariate *x* (set to 1 and 2) with specified coefficients, while non-significant variables are influenced by *x* with a coefficient set to zero. The coefficient for significant variables represents the effect size, determining how strongly the covariate *x* influences their outcomes. Larger absolute values indicate a stronger effect, making it easier to detect significant differences between groups. The study considers a sample size of 100, with 50 variables in total, of which 25 are significant. For significant variables, we use two different coefficients: β=−0.7 and β=−0.5. The reduction in the magnitude of the coefficient from -0.7 to -0.5 represents a decrease in effect size, making it harder to detect significant differences between groups. For non-significant variables, we set β=0, indicating no effect of the covariate *x* on these outcomes. Additionally, the intercept is set to be -2.

Zero-inflation is modeled by multiplying the original data in both groups with data simulated from a Bernoulli distribution based on the specified probability *q*, referring to the occurrence of excess zeros in the data beyond what is expected from the beta distribution alone. Higher values of *q* result in more zeros in the data, posing greater challenges in detecting significant differences. The simulation is performed for both values of *β* and for the same set of zero-inflation probabilities (q=0%,30%,50%,70%), where different values of *q* represent different scenarios: for example, q=0% may correspond to datasets aggregated to class level, while higher *q* values indicate increasing levels of zero-inflation typically found in species-level data. For details and the algorithm for this Zero Inflated Beta Regression Simulation, please refer to Supplementary Section 4.

Conversion methods applied to the simulated data include Log conversion, Logit conversion, Arcsine conversion, Box-Cox conversion, Tangent conversion, DGTL conversion, DGBC conversion. The two-sample t-test is used to assess the significance of differences between groups. Our motivation for using the two-sample t-test is that it assumes the data follow a normal distribution, allowing us to evaluate the effectiveness of the conversion methods in achieving this assumption. Additionally, the Wilcoxon rank-sum test [85] is used as a non-parametric reference method. The power, FDR, and their standard deviations are calculated for each transformation method.

Conversions like log, logit, Box-Cox, and DGBC cannot handle zeros, so we replace zeros with a very small number, $1\times 10^{-10}$. This replacement allows the transformations to be applied without encountering undefined values.

The results of our simulations, including the power, false discovery rate (FDR), and their standard deviations for each conversion method, are presented in Table 2. Several key observations can be made from the table regarding the effectiveness of different conversion.

**Table 2:** Power and false discovery rate (FDR) for various conversion methods in proportional data analysis under different conditions.

*β*	Conversion	Percent of 0							
		0%		30%		50%		70%	
		Power	FDR	Power	FDR	Power	FDR	Power	FDR
-0.7	No conversion	0.4172 ± 0.1038	0.0252 ± 0.0325	0.1884 ± 0.0763	0.0172 ± 0.0256	0.0976 ± 0.0543	0.0164 ± 0.0267	0.0368 ± 0.0354	0.0164 ± 0.0267
	Log conversion	0.8552 ± 0.0689	0.0336 ± 0.0363	0.2116 ± 0.0695	0.0312 ± 0.0329	0.1008 ± 0.0573	0.0344 ± 0.0386	0.0468 ± 0.0390	0.0296 ± 0.0358
	Logit conversion	0.8552 ± 0.0689	0.0344 ± 0.0369	0.2116 ± 0.0695	0.0312 ± 0.0329	0.1008 ± 0.0573	0.0344 ± 0.0386	0.0468 ± 0.0390	0.0296 ± 0.0358
	Arcsine conversion	0.6256 ± 0.0961	0.0316 ± 0.0352	0.2696 ± 0.0916	0.0224 ± 0.0292	0.1420 ± 0.0631	0.0220 ± 0.0292	0.0600 ± 0.0460	0.0208 ± 0.0303
	Box-Cox conversion	0.8340 ± 0.0729	0.0356 ± 0.0389	0.2384 ± 0.0786	0.0312 ± 0.0339	0.0992 ± 0.0587	0.0344 ± 0.0386	0.0408 ± 0.0355	0.0296 ± 0.0358
	Tangent conversion	0.0024 ± 0.0095	0.0020 ± 0.0088	0.0288 ± 0.0307	0.0260 ± 0.0308	0.0244 ± 0.0311	0.0328 ± 0.0391	0.0288 ± 0.0322	0.0296 ± 0.0358
	DGTL conversion	0.8552 ± 0.0689	0.0360 ± 0.0383	0.2956 ± 0.0927	0.0288 ± 0.0322	0.1520 ± 0.0675	0.0268 ± 0.0327	0.0644 ± 0.0462	0.0264 ± 0.0347
	DGBC conversion	0.8392 ± 0.0752	0.0356 ± 0.0389	0.2384 ± 0.0775	0.0312 ± 0.0339	0.0988 ± 0.0561	0.0344 ± 0.0386	0.0416 ± 0.0359	0.0296 ± 0.0358
	Wilcoxon Rank-Sum Test	0.8076 ± 0.0743	0.0336 ± 0.0372	0.2340 ± 0.0803	0.0276 ± 0.0315	0.0876 ± 0.0553	0.0280 ± 0.0319	0.0356 ± 0.0341	0.0292 ± 0.0363

-0.5	No conversion	0.2620 ± 0.0832	0.0144 ± 0.0231	0.0912 ± 0.0613	0.0144 ± 0.0264	0.0516 ± 0.0419	0.0152 ± 0.0233	0.0304 ± 0.0360	0.0116 ± 0.0215
	Log conversion	0.5336 ± 0.1005	0.0184 ± 0.0292	0.0684 ± 0.0503	0.0312 ± 0.0349	0.0624 ± 0.0449	0.0300 ± 0.0343	0.0364 ± 0.0390	0.0300 ± 0.0313
	Logit conversion	0.5336 ± 0.1005	0.0196 ± 0.0298	0.0684 ± 0.0503	0.0312 ± 0.0349	0.0624 ± 0.0449	0.0300 ± 0.0343	0.0364 ± 0.0390	0.0300 ± 0.0313
	Arcsine conversion	0.3824 ± 0.0979	0.0188 ± 0.0298	0.1232 ± 0.0723	0.0224 ± 0.0303	0.0764 ± 0.0502	0.0232 ± 0.0327	0.0420 ± 0.0456	0.0192 ± 0.0263
	Box-Cox conversion	0.5132 ± 0.0999	0.0208 ± 0.0309	0.0916 ± 0.0621	0.0320 ± 0.0350	0.0612 ± 0.0435	0.0296 ± 0.0339	0.0340 ± 0.0383	0.0300 ± 0.0313
	Tangent conversion	0.0032 ± 0.0109	0.0008 ± 0.0056	0.0224 ± 0.0303	0.0272 ± 0.0331	0.0332 ± 0.0346	0.0292 ± 0.0336	0.0300 ± 0.0338	0.0292 ± 0.0311
	DGTL conversion	0.5380 ± 0.1016	0.0212 ± 0.0309	0.1276 ± 0.0686	0.0268 ± 0.0337	0.0800 ± 0.0482	0.0236 ± 0.0301	0.0424 ± 0.0440	0.0236 ± 0.0312
	DGBC conversion	0.5200 ± 0.0988	0.0208 ± 0.0309	0.0884 ± 0.0626	0.0320 ± 0.0350	0.0612 ± 0.0435	0.0300 ± 0.0343	0.0340 ± 0.0383	0.0300 ± 0.0313
	Wilcoxon Rank-Sum Test	0.4972 ± 0.1038	0.0200 ± 0.0304	0.1004 ± 0.0632	0.0264 ± 0.0328	0.0628 ± 0.0452	0.0256 ± 0.0319	0.0360 ± 0.0392	0.0276 ± 0.0320

The DGTL conversion consistently demonstrates high power across various levels of zero-inflation while maintaining a low FDR, indicating robustness and reliability. The Logit and Log conversion also exhibit relatively high power, but only when zero-inflation is low. The Arcsine conversion performs inadequately when zero-inflation is 0%, but as the percentage of zeros increases, its power becomes more competitive, ranking just behind the DGTL conversion at 70% zero-inflation, which also makes it a stable choice when zero-inflation is high. The Tangent conversion generally shows the worst power, indicating it is not suitable for this analysis.

Beyond comparing the power of each conversion method, analyzing their skewness and kurtosis is important to determine which one has the highest conversion ability. Our table in Supplementary Section 5 provides a comprehensive summary of skewness and kurtosis statistics for various conversions applied to datasets with different *β* values and percentages of zero values.

The dual group conversion, specifically the DGTL and DGBC conversion, generally outperform their traditional counterparts. Dual group conversion not only helps transform the data to a normal distribution using optimization but also has better efficiency in preserving signals after conversion. Although the DGTL conversion does not always result in significant improvements in conversion compared to the traditional logit conversion, it consistently demonstrates better power and relatively lower false discovery rate. Table 2 further supports this by showing the enhanced power of the DGTL conversion, which is notably higher than that of the traditional logit conversion, indicating its superior ability to detect true effects in the data, especially in the presence of zeros.

Besides understanding which conversion has higher power, managing outliers is another critical aspect that requires our attention. Outliers often result from variations in sample collection, processing, sequencing, and biological differences between individuals. These discrepancies introduce significant noise, which can overshadow genuine biological signals and negatively impact the effectiveness and accuracy of analysis [25], [48], [86], [87]. Therefore, we conducted a detailed simulation study. The goal was to compare the performance of different traditional conversions (Log, Logit, Arcsine, Box-Cox) under various conditions. Using simple linear regression, the simulations involved generating data with different $\beta_{0}$ values and adding random noise uniformly distributed in the range from -0.18 to 0.18. Three scenarios were considered: no outliers, left outliers (smaller outliers: 0.1, 0.01, 0.001, 0.0001, 0.00001, 0.000001), and right outliers (larger outliers: 0.9, 0.99, 0.999, 0.9999, 0.99999, 0.99999). For each scenario, intercepts and standard errors were estimated using linear models. The mean and standard error of the intercept estimates were calculated across 100,000 simulations for each $\beta_{0}$ value, and the results were compiled into a combined plot to visualize the performance of each conversion in managing variance and handling outliers.

**Fig. 3:**
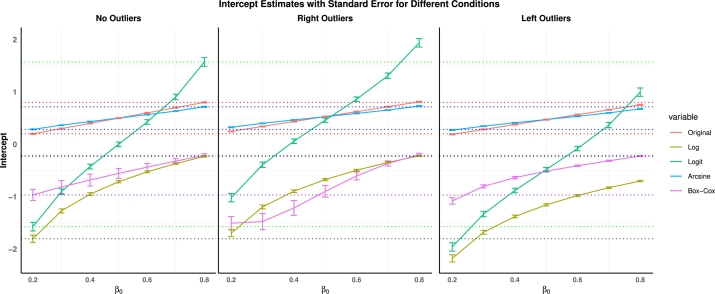
Intercept and Standard Error Estimates for Different Conditions (No Outliers, Right Outliers, Left Outliers). The plots compare the performance of different conversion (Original, Log, Logit, Arcsine, Box-Cox) in terms of their intercepts and standard errors across various *β*_0_ values. Right outliers represent larger outliers (0.9, 0.99, 0.999, 0.9999, 0.99999, 0.99999), while left outliers represent smaller outliers (0.1, 0.01, 0.001, 0.0001, 0.00001, 0.000001). The results highlight the robustness of the arcsine conversion in managing variance and handling outliers.

The analysis depicted in Fig. 3 provides several critical insights into the effectiveness of different conversion methods in managing variance and handling outliers. Log conversion is particularly sensitive to left outliers (smaller outliers) but is less sensitive to right outliers. However, it tends to expand the variance compared to the original data. Logit conversion is sensitive to both right and left outliers and also fails to control variance effectively. Box-Cox conversion, which selects the parameter *λ* based on the data, is highly influenced by right outliers and less so by left outliers, and similarly cannot control variance. This contradicts the common belief that log, logit, and Box-Cox conversion can reduce variance and mitigate the influence of outliers.

Notably, for Box-Cox conversion, the influence of right outliers is particularly severe when $\beta_{0}$ is small. As $\beta_{0}$ increases, the influence of right outliers becomes relatively smaller. Additionally, the standard error for log and Box-Cox conversions is reduced as $\beta_{0}$ increases. However, it is important to highlight that for microbiome data, which is typically compositional and right-skewed, these conversion methods may not be as effective in reducing variance and handling outliers due to the inherent characteristics of the data.

In contrast, the arcsine conversion demonstrates robustness to outliers. The intercepts for the arcsine-converted data remain relatively stable, even in the presence of outliers. Additionally, the standard errors for the arcsine conversion are consistently lower, indicating that it effectively reduces variance.

### 2.4 Contrast transformations for compositional data

Compositional data were defined traditionally as constrained data with a fixed constant sum constraint (1 or 100) [88]. The microbial sequence read counts carry relative information, because the total number of counts is fixed and different across samples. TSS transforms them to relative abundances, imposing the simplex constraint where the components sum to one. Thus, the degree of freedom is reduced by one. The unit-sum constraint can induce spurious correlations among components, complicating the interpretation of statistical measures such as correlation and variance [22]. This inherent interdependence poses unique challenges for statistical analysis since traditional multivariate techniques, designed for unconstrained data, can produce misleading results when applied to compositional data [22], [23].

Appropriate transformations are essential to preserving the same degree of freedom for both the original and transformed data, and to improving the properties of the transformed data by relaxing the simplex constraint. To address these challenges, John Aitchison laid the groundwork for compositional data analysis (CoDA) by developing methods that respect the relative nature of compositional data. He introduced the concept of log-ratios to handle compositional data appropriately, arguing that the ratios between components are more meaningful than their absolute values [89]. This approach transforms the data out of the simplex, breaking the sample space of the compositional data out of a constrained hyperplane and into the real vector space. This transformation allows for the application of standard statistical techniques while maintaining the relative nature of the data, as the product of a log ratio is transformed to real space, making the data appear independent [90], [91].

We derive the contrast transformation from the log-ratio transformation by omitting the univariate log conversion. For now, we focus on contrast transformation and we give its definition as follows. A contrast transformation for compositional data is a linear transformation used to analyze the relative differences between components in a composition, while respecting the inherent sum constraint of the data (i.e., the components sum to a constant, typically 1). In contrast transformations, each contrast is constructed to compare parts of the composition, ensuring that the sum of the coefficients for each component in the contrast equals zero. This approach eliminates the influence of the total sum or size, focusing solely on the relative relationships between the components. Let $C=(c_{1},c_{2},\ldots ,c_{p})$ represent a p×p dimensional contrast transformation matrix, where each column vector is orthogonal to the vectors of one's, denoted by **1**, implying that $c_{j}^{T}1=0$. The orthogonality is necessary to transform the simplex into a new space that is uncorrelated with the original simplex. Based on this simple and general condition, various contrast transformations can be designed. Several well-known examples and realizations are provided below.

#### 2.4.1 Additive contrast (AC)

Based on the comprehensive summary of compositional data analysis by Greenacre [92] and the detailed demonstration of Supplementary materials by Zhang et al. [93], the additive contrast matrix is defined by

(1) $$C={\left(\begin{array}{cccccc} 1 & 0 & 0 & \cdots & 0 & 0\\ 0 & 1 & 0 & \cdots & 0 & 0\\ 0 & 0 & 1 & \cdots & 0 & 0\\ \vdots & \vdots & \vdots & \ddots & \vdots & \vdots \\ 0 & 0 & 0 & \cdots & 1 & 0\\ -1 & -1 & -1 & \cdots & -1 & 0 \end{array}\right)}_{p\times p},=I_{p\times p}-{\left(0_{p\times (p-1)};1_{p}\right)}_{p\times p}^{T},$$

where $I_{p\times p}$ denotes a *p*-dimensional diagonal matrix and ${\left(0_{p\times (p-1)};1_{p}\right)}_{p\times p}^{T}$ denotes a *p*-dimensional matrix with the last row consisting of 1's and all other elements set to 0.

Here is an intuitive explanation. Multiplying the data by the AC matrix ***C*** means that each component of a sample is subtracted by the last component. We can modify the position of the row of 1's in ${\left(0_{p\times (p-1)};1_{p}\right)}_{p\times p}^{T}$. If the 1's are placed in the *j*-th row, then the *j*-th component is chosen as the reference. The last column of the matrix ***C*** contains only zeros, because the reference component is subtracted from itself. This omission results in the transformed data having only p−1 columns, thus preserving the same degrees of freedom as the original compositional data. Researchers typically remove the last column of ***C*** as it does not affect the calculation. Utilizing the resulting p×(p−1) matrix simplifies the transformation process.

#### 2.4.2 Centered contrast (CC)

Similarly, the centered contrast matrix is defined by

(2) $$\begin{array}{cc} C & ={\left(\begin{array}{ccccc} 1-\frac{1}{p} & -\frac{1}{p} & -\frac{1}{p} & \cdots & -\frac{1}{p}\\ -\frac{1}{p} & 1-\frac{1}{p} & -\frac{1}{p} & \cdots & -\frac{1}{p}\\ -\frac{1}{p} & -\frac{1}{p} & 1-\frac{1}{p} & \cdots & -\frac{1}{p}\\ \vdots & \vdots & \vdots & \ddots & \vdots \\ -\frac{1}{p} & -\frac{1}{p} & -\frac{1}{p} & \cdots & 1-\frac{1}{p} \end{array}\right)}_{p\times p}\\ & =I_{p\times p}-\frac{1}{p}{\left(1^{T}1\right)}_{p\times p} \end{array}.$$

Multiplying the data by the CC matrix ***C*** subtracts the average from each component of a sample. The centered contrast transformation treats all components symmetrically, but it introduces a new constraint: the sum of the transformed components is zero [94]. This means that the transformed sample lies on a plane passing through the origin of $R^{D}$, enabling the use of standard statistical techniques in Euclidean space. Additionally, this transformation preserves the degrees of freedom at p−1, maintaining consistency with the original sum-constrained data.

Beyond the additive and centered contrasts discussed, other contrast transformations, such as pairwise contrast (employed in pairwise logratios) [92] and pivot contrast (utilized in pivot logratios) [88], [92], are also commonly used. Additionally, nonlinear contrasts such as amalgamation (or summated) contrast offer another approach to compositional data analysis [92], [95]. However, due to space constraints, this paper focuses primarily on the additive and centered contrasts.

Contrast transformations are not exclusive to compositional data analysis; they are widely used in the context of ANOVA and regression models to test specific hypotheses about group means. This application predates their use in compositional data analysis, with the theory behind ANOVA formalized by Ronald Fisher in the 1920s. A key reference for understanding contrast coding and transformations in statistical models is Kutner et al. [96], which offers a detailed explanation of contrast coding and transformations in linear models. The book covers important concepts such as orthogonality and how contrast transformations facilitate comparisons of group means.

### 2.5 Revamp compositional data transformation

Suppose we have an n×p compositional data matrix $X=(x_{1},x_{2},\ldots ,x_{p})$, where each column vector $x_{j}$ (for j=1,2,…,p) denotes the *j*-th variable. Without loss of generality, we assume that each row of ***X*** lies on a simplex, where $x_{ij}>0$ and $\sum_{j=1}^{p}x_{ij}=1$ for i=1,2,…,n. This structural property reduces the degrees of freedom of the data matrix to p−1. The right multiplication of a contrast matrix ***C*** defines the application of a contrast transformation. The transformed data can be represented as

$$XC=\left(\sum_{j=1}^{p}x_{ij}c_{jk}\right),i=1,2,\ldots ,n;k=1,2,\ldots ,p,$$

where $c_{jk}$ are the contrast coefficients, which satisfy $\sum_{j=1}^{p}c_{jk}=0$. Therefore, each contrast actually extracts relative information and compares the parts of the compositions.

Microbial relative abundance is both proportional and compositional. Following Aitchison [89]'s seminal work on log-ratio transformations, a similar analogy for compositional data transformation typically involves two steps: first, applying conversion to the proportions, and then performing a contrast transformation. Based on this understanding, we propose a new framework of compositional data analysis that combines univariate proportion conversion and multivariate contrast transformation (as shown in Fig. 1). We call this the CCT (Conversion and Contrast Transformation) framework. In this framework, we use *g* to represent a conversion function for proportional data. Afterwards, we apply the right multiplication of a contrast matrix ***C***. Then the framework of compositional data transformation can be defined as

(3) T=g(X)C.

Within this framework, two commonly used methods, ALR and CLR, are special cases. This section reviews these two methods, along with other established methods, laying the groundwork for the novel transformations proposed in subsequent sections. By revisiting these classical approaches, we aim to highlight both their strengths and areas where innovation can further enhance their utility.

#### 2.5.1 Additive log ratio transformation

**Fig. 4:**
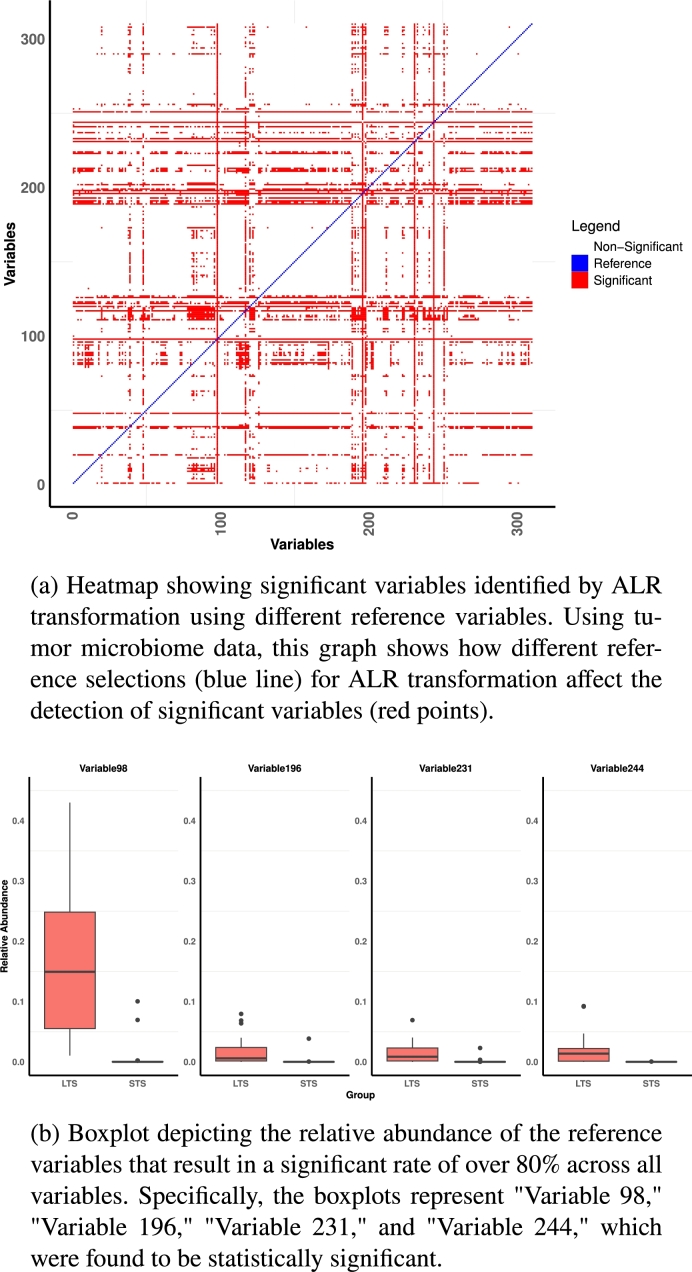
Effects of reference variable selection on ALR transformation.

If we use the **log** function to convert compositional data ***X*** and then multiply it by the additive contrast matrix ***C*** as defined in Equation (1), the application of Equation (3) yields the **ALR transformation** as a special case in this new framework. The *j*-th column of the transformed data can be calculated as

$${\text{ALR}}_{j}=\log(X)c_{j}=\log(x_{j})-\log(x_{D})=\log\left(\frac{x_{j}}{x_{D}}\right),\text{for }j=1,2,\ldots ,p,$$

where $x_{D},D\in \{1,2,\ldots ,p\}$ represents a chosen reference. Introduced by Aitchison [22], the ALR transformation has several advantages, including simplicity and ease of interpretation, especially when the reference component is biologically or chemically meaningful. For example, in microbiome studies, a stable and ubiquitous microbial species can serve as a reference, providing clear and interpretable results [97]. However, choosing different references may cause totally different results [89], [98]. We ran a real data study to show the significant changes in abundance tests. As shown in Fig. 4a, we conducted two group t-tests on pancreatic tumor microbiome data [9] to evaluate the impact of selecting different references on the ALR transformation and differential analysis. Before we use the ALR transformation, we also filter the taxa. Initially, the data consisted of 2288 taxa, and we filtered out those taxa where more than 90% of the data were zeros, leaving us with 310 taxa. Both x-axis and y-axis denote the variable positions in the data. The blue diagonal line in the figure represents the chosen reference, moving from the first to the last position in the data. The red dots along the y-axis indicate the variables identified as significant. In other words, the x-axis corresponds to the variables chosen as references, and a vertical examination reveals which variables become significant for each specific reference.

Horizontal red lines imply that these variables are consistently identified as significant regardless of the reference chosen. Vertical red lines indicate choosing these references result in the majority of the variables being identified as significant. We isolated references that cause a significant rate of over 80% across all variables. Specifically, “Variable 98” resulted in 100% of variables being significant, “Variable 196” resulted in 96.4% of variables being significant, “Variable 231” resulted in 89.6% of variables being significant, and “Variable 244” resulted in 97.4% of variables being significant. We created boxplots for these four references in Fig. 4b and found them significantly differential between two groups. This result indicates that using different references leads to dramatically inconsistent testing outcomes. Utilizing highly significant references may produce lots of false positives.

Outliers in the reference (as shown in Fig. 4b) can potentially have a strong impact on ALR transformation. To investigate this, we removed the outliers and retested, creating a new figure (similar to Fig. 4a) in our paper's Supplementary material section 2. We used interquartile range (IQR) filtering method [99] to remove outliers by filtering them based on log-converted values. Specifically, we log-converted the non-zero values, calculated the IQR, and then filtered out values outside 1.5 times the IQR from the first and third quartiles. We found that outliers negatively influenced detection. Additionally, using different values to replace zeros in ALR transformation also impacted differential abundance detection, which we illustrated in Fig. S1 in Supplementary material Section 2.

In general, for the ALR transformation, choosing a reference is crucial. We recommend choosing a reference that is not significant and has little or no outliers. Moreover, selecting a value to replace zeros is important and needs careful consideration. As mentioned by Greenacre et al. [100], the reference can be chosen to maximize the Procrustes correlation between the additive logratio geometry and the exact logratio geometry, as well as to minimize the variance of the reference component's log-transformed relative abundance values, making the subsequent interpretation of the logratios even easier [100]. Additionally, it is important to avoid references with low abundances or many zeros, as replacing zeros can impact the interpretation of ALRs and zeros cannot provide information, making it challenging to draw meaningful conclusions from them [100].

Additionally, ALR transformation sacrifices one component to serve as the denominator, and the transformed variables are not isometric, meaning they do not preserve the original geometric relationships exactly. These limitations are often acceptable in practice, given the benefits of simplicity and interpretability [22], [90]. Another significant issue is the presence of zeros in the data, which can complicate the transformation and subsequent analysis. Various strategies, such as zero replacement or imputation, have been proposed, but they can introduce biases and affect the robustness of the results [22].

#### 2.5.2 Centered log ratio transformation

If we use the **log** function to convert compositional data ***X*** and then multiply it by the centered contrast matrix ***C*** as defined in Equation (2), the application of Equation (3) yields the **CLR transformation** as a special case in this new framework. The *j*-th column of the transformed data can be calculated as

$${\text{CLR}}_{j}=\log(X)c_{j}=\log(x_{j})-\frac{1}{p}\log(\sum_{j=1}^{p}x_{j})=\log\left(\frac{x_{j}}{f(X)}\right),\text{for}\;j=1,2,\ldots ,p,$$

Mathematically, f(X) is the geometric mean of the components of ***X***, defined as $f(X)={\left(\prod_{j=1}^{p}x_{j}\right)}^{1/p}$.

CLR is another fundamental technique in the analysis of compositional data introduced by Aitchison [22]. This transformation projects the compositional data into a higher-dimensional space where the components sum to zero (hyperplane passing through origin), ensuring that the data is appropriately scaled and enabling the application of Euclidean geometry [22], [90].

The CLR transformation has several advantages. Unlike ALR, CLR is invariant to the choice of reference. The geometric mean transformation ensures that the results are not affected by the selection of any particular component as the reference [101]. It often yields a more normal-like data distribution by centering around the geometric mean. It preserves the relative information among components, ensuring that no single component is disproportionately weighted or treated differently from others.

However, the CLR transformation is not without challenges, particularly its sensitivity to zeros, as log conversion is undefined for zero values. Therefore, the CLR transformation requires all components to be non-zero [102]. Zero-replacement techniques, although helpful, can introduce biases and affect the analysis's robustness [13], [103]. CLR transformation can smooth out variability across components by centering around the geometric mean. This may lead to a loss of important variability information in the data. Additionally, the transformed variables sum to zero, resulting in collinear data and an incomplete solution to the constant sum constraint problem, as the data matrix remains not full rank [104], [105].

While ALR and CLR transformations are well-established and widely used in compositional data analysis, some alternative transformations like the Isometric Log-Ratio [106], *α*-transformation [107] and the Box-Cox transformation for compositional data [108] offer additional flexibility and advantages in specific scenarios. Each of these transformations extends the traditional log ratio methods by introducing different perspectives on orthonormality, normality, and optimality.

#### 2.5.3 Other transformations

Let's view our proposed CCT framework from another perspective: it begins with an initial conversion of each component, followed by the application of contrasts on each compositional vector. These contrasts represent linear transformations, forming overall linear combinations of converted components. With this in mind, we can examine whether other existing transformations can be incorporated into our CCT framework. The isometric log-ratio transformation [106] fits within our framework, as it can be viewed as the CLR transformation followed by multiplication with an additional orthogonal contrast matrix, which remains linear combinations of converted components. In contrast, transformations such as the *α*-transformation [107], [109] and the Box–Cox transformation for compositional data [108] fall outside the scope of our framework. Both involve taking ratios between components, which can not be expressed as linear combinations of converted components. These nonlinear approaches offer unique lens for compositional data transformations, and we explore them in more detail below.

The **Isometric Log-Ratio (ILR) transformation** is a robust method for compositional data analysis, introduced by Egozcue et al. [106], which preserves the geometric properties of the original data in the simplex by ensuring that distances and angles are maintained [110], [111]. Mathematically, it uses an orthonormal basis in the simplex to map compositional data to real space, which is defined using orthonormal basis vectors $e_{1},e_{2},\ldots ,e_{p-1}$:

$$\text{ILR}(X)=\left(\langle X,e_{1}\rangle ,\langle X,e_{2}\rangle ,\ldots ,\langle X,e_{p-1}\rangle \right),$$

where 〈⋅,⋅〉 denotes the inner product [106]. Additionally, the ILR transformation can be represented as ILR(X)=CLR(X)⋅H, where *H* is an orthonormal contrast matrix of dimensions p×(p−1), with rows that are orthogonal to the vector of ones, $1_{p}$. A common choice for *H* is the transposed Helmert sub-matrix, which is derived by removing the first row from the Helmert matrix [107], [112], as the Helmert matrix shown in the Supplementary material Section 6.

However, like ALR and CLR transformations, ILR is also sensitive to zeros. Additionally, selecting an appropriate orthonormal basis is crucial, as different bases can lead to varying representations [101]. While ILR preserves geometric structure [106], it may have lower statistical power compared to ALR and CLR, particularly with high-dimensional data or small sample sizes due to the orthonormal basis selection and transformation process. Despite these challenges, the ILR transformation remains valuable for its geometric consistency and effectiveness in compositional data analysis [106].

The *α***-transformation** for compositional data generalizes traditional log-ratio transformations. Because the logarithm transformation is a specific case of the power transformation when the power parameter *α* equals zero [107], [109]. To maintain consistency with the original literature, we use *D* instead of *p* to describe the dimension here. The transformation is defined as

$$z_{\alpha }(x)=H\cdot \left(\frac{Du_{\alpha }(x)-1_{D}}{\alpha }\right),$$

where α>0, $u_{\alpha }(x)$ is the compositional power transformation, $1_{D}$ is a vector of ones, and *H* is a matrix of orthonormal rows that are orthogonal to $1_{D}$. The power transformation $u_{\alpha }(x)$ is given by

$$u_{\alpha }(x)={\left(\frac{x_{1}^{\alpha }}{\sum_{j=1}^{p}x_{j}^{\alpha }},\ldots ,\frac{x_{p}^{\alpha }}{\sum_{j=1}^{p}x_{j}^{\alpha }}\right)}^{T}.$$

When *α* is set to 0, the transformation behaves as a log-ratio transformation, which is equivalent to performing log-ratio analysis. When *α* is set to 1, it functions as a linear transformation of the data, particularly when applied with discriminant analysis and nearest-neighbor classification methods, corresponding to Euclidean data analysis [109].

This transformation is advantageous for its flexibility, handling zeros and optimizing criteria like cross-validation in classification tasks, making it suitable for various fields such as geology, biology, and economics [109]. However, a disadvantage is its mapping to a subset of $R^{D-1}$, potentially ignoring probabilities outside the simplex. The **folded** *α***-transformation** addresses this by folding values back into the simplex, improving fit and applicability, though it increases computational complexity and lacks a one-to-one inverse transformation [113].

The **Box-Cox transformation for compositional data**, as described by Rayens and Srinivasan [108], enhances the traditional log-ratio approach by incorporating the Box-Cox family of transformations to achieve better normality in the transformed data. This transformation involves a two-step process where compositional data are first transformed into ratios and then subjected to a Box-Cox transformation. The ratios are formed as $y_{j}=\frac{x_{j}}{x_{p}}$ for j=1,2,…,p−1, the divisor $x_{p}$ is chosen without loss of generality (Rayens and Srinivasan [108]). The Box-Cox transformation is then applied to each ratio $y_{j}$:

$$BC(y_{j};\lambda_{j})=\left\{ \begin{array}{cc} \frac{y_{j}^{\lambda_{j}}-1}{\lambda_{j}} & \text{if }\lambda_{j}\neq 0\\ \log(y_{j}) & \text{if }\lambda_{j}=0 \end{array}\right.$$

The parameter $\lambda_{j}$ is chosen to best fit the data to a normal distribution [108]. This transformation generalizes ALR and allows for further extensions, as it includes the logarithmic transformation as a special case when $\lambda_{j}=0$.

The main advantage of using the Box-Cox transformation in this context is its ability to improve the fit to normality beyond what is achievable with a simple log conversion. However, a limitation of the Box-Cox transformation is that if different $\lambda_{j}$ parameters are used for different columns of the ratio-transformed data, it may change the covariance and compositionality among the columns of the original data. This could potentially mislead subsequent analyses. Additionally, like many traditional methods, the Box-Cox transformation for compositional data cannot handle zero values.

### 2.6 Novel transformations for compositional data

Statisticians have employed log-ratio transformations to handle microbiome data because it is compositional in nature. However, log-ratio transformations were not originally designed for data with a high prevalence of zeros, making them less appropriate for microbiome datasets. In fields like material science, chemistry, or ecology, zero inflation was not a significant issue in compositional data [52]. With the advent of omics data, particularly since the Human Genome Project launched in 1986, the presence of excess zeros has become more common, posing additional challenges in sequencing data analysis.

When performing log-ratio transformations, a common strategy to handle zeros is to replace them with a small value (e.g., 0.5 in count data). However, this approach introduces bias and may distort the results [22], [76], [114]. To briefly demonstrate these issues, we defined a group effect and conducted a simulation using zero-inflated negative binomial (ZINB) models [115] by varying the percentage of zeros. We then applied different constants for zero-replacement and used both ALR and CLR transformations. For each transformed dataset, we performed t-tests to assess the power and false discovery rate (FDR) in differential testing. A two-way ANOVA was conducted to investigate the impact of zero-inflation and zero-replacement on both power and FDR. Ideally, power and FDR should remain consistent, but all resulting p-values are significant, indicating that both the proportion of zeros and the choice of values for zero-replacement have a substantial impact on the statistical significance of the tests. Further details are provided in Supplementary Section 11.

This inconsistency and distortion motivated us to replace the log function in log-ratio transformations. Therefore, we propose the arcsin transformation as an alternative. It is well-defined at zero and does not require zero-replacement, making it a more suitable option.

#### 2.6.1 Developed new transformations within this framework

**Fig. 5:**
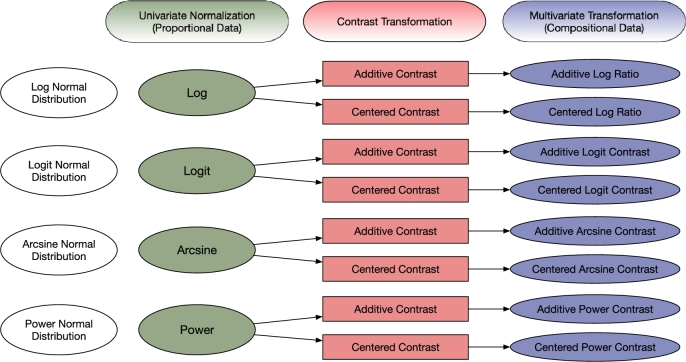
Diagram of framework for developing new compositional data transformations. This framework integrates proportion conversion with contrast transformation to create innovative transformation methods.

Among the various extensions and options discussed, we focus on our proposed CCT framework for developing new compositional data transformations. Fig. 5 elucidates some existing and newly developed transformations. This framework integrates univariate conversion of proportions with contrast transformations for compositions. The univariate conversion on the left stabilizes variance, manages zeros, and mitigates the impact of outliers. The contrast transformation in the middle releases the simplex constraint while preserving the degrees of freedom. The multivariate transformation on the right represents the newly developed compositional data transformations. This figure illustrates just a few examples of combinations, but it actually opens up a wide range of possibilities for researchers.

For compositional data *X*, the Additive Arcsine Contrast (AAC) for the *j*-th component is defined as:

$${\text{AAC}}_{j}=\text{arcsine}(X)c_{j},$$

for j=1,2,…,p, where ***C*** is the additive contrast matrix as defined in Equation (1). This approach demonstrates the versatility of the framework in applying the arcsine transformation in combination with additive contrast.

Notably, compared with log conversion, arcsine conversion stands out as it effectively handles boundary values (0 and 1) without the need for zero replacement, thereby reducing bias and enhancing robustness. As shown in Section 2.3.7, the arcsine conversion also stabilizes variance and controls outliers more effectively than log, logit, or Box-Cox transformations.

The choice between contrast transformations, such as AC or CC, represents another important consideration in ensuring the accuracy of compositional data analysis. Both AC and CC transformations aim to shape the data, promoting symmetry. When choosing between these transformations for compositional data analysis, it is essential to consider the structure and complexity of the data. This consideration is similar to selecting between Additive Logratio (ALR) and Centered Logratio (CLR) transformations after deciding to use log conversion as the univariate conversion.

The AC is particularly suitable for simpler data structures where a natural reference part exists. It reduces the dimensionality by one, making it more straightforward to interpret in models with fewer parameters. This approach is beneficial in regression and classification tasks where comparisons relative to a specific reference are meaningful [116], [117], [118]. AC is frequently employed when researchers are specifically interested in a particular taxon, as it allows for direct comparison against a chosen reference component, making it valuable in microbiome studies focusing on a single taxonomic group.

On the other hand, the CC is preferred for more complex data structures where no single reference part is appropriate. It maintains the full dimensionality, providing a balanced representation of all components. This method is optimal for exploratory data analysis, Principal Component Analysis (PCA), and other multivariate techniques where gaining insights into the overall data structure is essential [22], [105], [117].

Based on the Fig. 5, the transformations we proposed include Additive Power Contrast (APC), Additive Logit Contrast (ALTC), Additive Arcsine Contrast (AAC), Centered Power Contrast (CPC), Centered Logit Contrast (CLTC), and Centered Arcsine Contrast (CAC). Additionally, we have combined the new proposed DGTL and DGBC proportional data conversion in Section 2.3.6 with contrast transformations. This results in new transformations such as Additive Dual Group Truncated Logit Contrast (ADGTLC), Additive Dual Group Box-Cox Contrast (ADGBCC), Contrast Dual Group Truncated Logit Contrast (CDGTLC), and Contrast Dual Group Box-Cox Contrast (CDGBCC). Details are provided in Supplementary Material Section 12.

Moreover, these proposed transformations pave the way for many additional compositional data transformations through the combination of various proportional data conversion methods with contrast transformations. Researchers can further explore and propose new combinations, enhancing the flexibility and applicability of compositional data analysis techniques in diverse fields.

## 3 Evaluating compositional data transformations through simulation

To thoroughly evaluate the performance of various compositional data transformations, we conducted extensive simulations using two primary methods: the Zero Inflated Negative Binomial (ZINB) regression model [115] and the SimulateMSeq function from the GUniFrac package [119]. These simulations are designed to mimic real-world scenarios in microbiome research, where data often exhibits complex characteristics such as zero inflation, dispersion, and varying sequencing depths.

### 3.1 Simulation with zero inflated negative binomial model [115]

We simulated microbiome count data using a zero-inflated negative binomial regression model [115], as this approach provides better control over zero-inflation. Then we conducted various transformations to the relative abundances. For differential testing, we mostly relied on a two-sample t-test, which assumes that the data follow a normal distribution, as the goal of these transformations is to achieve a more normal-like data distribution. Our decision to employ fundamental tests, such as the t-test and Wilcoxon rank-sum test, stems from their simplicity, widespread use, and adaptability to more advanced methods like regression. Our goal was to demonstrate that if these basic tests perform well with our proposed transformations, then more sophisticated tests would also be applicable and potentially even more effective.

The simulation was conducted with the following parameters: a sample size of 100, 50 variables with 25 significant. The significant variables were influenced by the covariate *x*, which equals to 1 and 0, and the significant coefficient *β* ranging from 1 to 9 by increments of 2. The higher the coefficient, the more significant the variable. While the non-significant coefficient *β* is zero, ensuring they are generated from the same distribution. The dispersion parameter *α* ranging from 1 to 9 by increments of 2, the intercept $\beta_{0}$ ranging from 1 to 9 by increments of 2, and zero-inflation probabilities *q* ranging from 0 to 0.8 by increments of 0.2. Zero-inflation is applied to both groups, with zeros generated from the Bernoulli distribution using a probability of zero, *q*. Each parameter combination underwent 100 independent simulation runs to ensure robustness. Details of the full methodology and the algorithm for this zero-inflated negative binomial regression simulation can be found in Supplementary Section 7.

Because some transformations method cannot handle zeros, we replace zeros in the count data for these transformations with 0.5 before TSS. This replacement allows the transformations to be applied without encountering undefined values. Specifically, we replace zeros for the following transformations: ALR, CLR, APC, CPC, ALTC, CLTC, ADGBCC, CDGBCC, Box-Cox in Ratio, Dual Group Box-Cox in Ratio, and ILR.

The results of our simulations, including the coefficients for each transformation method derived from the linear regression model, are presented in Table 3. By training a linear regression model with mean power as the dependent variable and the transformation parameters (*α*, $\beta_{0}$, *β*, and *q*) as the independent variables, we can directly assess the contribution of each parameter to the transformation's performance. This method allows us to identify which transformations are most effective under varying conditions, providing a clear advantage over traditional methods that do not account for these nuances. The intercept represents the baseline power for each transformation method, which is crucial for understanding the inherent effectiveness of each method.

**Table 3:** Power comparisons of different transformations under various conditions using data simulated by a ZINB model. The coefficients indicate the impact of diverse data characteristics on the testing power of each method. The parameters are defined as follows: the intercept represents the baseline power for each transformation method. The *α* coefficient indicates how dispersion affects power, *β*_0_ shows the effect of the regression model intercept on power, *β* represents the influence of effect size, and *q* reflects the impact of zero-inflation probability on power.

Transformation	Intercept	*α*	*β*_0_	*β*	*q*
ALR	0.5219	-0.0223	-0.0267	0.0733	-0.8272
CLR	0.3625	-0.0243	-0.0100	0.0367	-0.5650
ALTC	0.5240	-0.0225	-0.0265	0.0733	-0.8292
CLTC	0.3631	-0.0244	-0.0099	0.0366	-0.5643
AAC	0.5928	-0.0559	0.0014	0.0424	-0.6773
CAC	0.2654	-0.0293	0.0006	0.0133	-0.2758
APC	0.5711	-0.0299	-0.0210	0.0800	-0.7818
CPC	0.5211	-0.0380	-0.0137	0.0715	-0.6815
ADGTLC	0.5603	-0.0494	0.0020	0.0571	-0.7629
CDGTLC	0.3332	-0.0360	0.0023	0.0222	-0.4025
ADGBCC	0.3391	-0.0130	-0.0138	0.1015	-0.5881
CDGBCC	0.2351	-0.0089	-0.0118	0.0967	-0.4925
ILR	0.0223	-0.0005	-0.0017	0.0044	-0.0242
Box-Cox in Ratio	0.5047	-0.0229	-0.0192	0.0672	-0.8462
Dual Group Box-Cox in Ratio	0.5268	-0.0234	-0.0235	0.0716	-0.8490

We also created a linear regression model for FDR, using the same approach as the linear regression for power. The detailed results are provided in the Supplementary Section 8. Notably, the FDR for most of the transformations is inversely correlated with power, meaning that higher power for a transformation corresponds to a lower FDR. In particular, the coefficient of *q* value in the FDR table for the AAC is 0.021137927, the smallest among the transformations, indicating that an increase in the percentage of zeros in the data frame has the relatively smallest impact on its FDR.

Among the various transformations evaluated, the AAC stands out with the highest intercept of 0.5928, indicating superior baseline power for compositional data analysis, especially in zero-inflated datasets. Its effectiveness is due to the arcsine normal distribution, where *y* approaches infinity as *x* approaches 0, unlike traditional distributions like the logit normal distribution. However, in scenarios with less severe zero inflation, other transformations may outperform the AAC.

The APC also demonstrates significant baseline power with an intercept of 0.5711, making it a robust option for compositional data. This transformation's flexibility is balanced by the potential issue of different variables choosing different *λ* values, which can disrupt variable correlations.

The ADGTLC shows strong baseline power with an intercept of 0.5603689938, providing a viable alternative to the AAC, but shares the same issue as the APC.

Other methods like the ALTC and Dual Group Box-Cox in Ratio also demonstrate strong baseline power with intercepts of 0.5240 and 0.5268, respectively, offering reliable alternatives for researchers.

The ILR method demonstrates the lowest intercept at 0.0223, suggesting it may not be suitable for achieving high power in compositional data analysis.

However, Centered Contrast transformation methods like the CLR have a lower intercept of 0.3625416, indicating lower baseline power. Additionally, methods like CDGBCC and CAC exhibit even lower baseline power compare with Additive Contrast transformation methods. This result contradicts the common belief that CLR transformation is generally preferred. Aitchison's recommendation of the CLR transformation underscores this belief. Additionally, this method has gained traction in the microbial literature, where it has been argued that the CLR transformation can effectively analyze microbiome data, RNA-seq data, and any next-generation sequencing data set [97], [120]. Moreover, the CLR transformation is the most widely used and convenient for compositional data [10].

Our simulation serves as an illustrative example demonstrating that researchers cannot arbitrarily choose between AC methods like ALR and CC methods like CLR. The choice between these methods is highly dependent on the data structure, necessitating a thorough exploration of the data prior to analysis. Although CC transformations like CLR are generally more robust and less sensitive to outliers, our simulation is a good example showing a situation where AC is better than CC. In count data, particularly in microbiome studies with multiple groups such as cancer patients and healthy controls, if variables in one group consistently show higher or lower counts compared to another group, using AC transformations (such as the ALR) becomes more favorable over CC transformations (such as the CLR).

The CLR transformation averages all variables, which can diminish the signal of significant variables. This may result in the dilution of the impact of truly significant changes, while also amplifying noise. Consequently, this can introduce false signals to non-significant variables, thereby reducing statistical power and increasing the false discovery rate (FDR). In contrast, ALR transformation compares variables directly to a chosen reference, preserving the relative differences between groups more effectively. Therefore, when analyzing microbiome count data with distinct group differences, ALR is a preferable transformation method to CLR. However, when the dataframe is complex and trends are difficult to discern, CLR transformation can also be a good choice, as it can help to standardize the data and reveal underlying patterns.

In conclusion, the AAC and APC exhibit significantly better power compared to other transformations. The AAC is highly effective for zero-inflated data, while the APC offers a robust alternative, though care must be taken with variable *λ* values to avoid disrupting data associations. These findings underscore the importance of selecting appropriate transformation methods with high intercepts to ensure accurate and reliable results in microbiome research. Furthermore, the choice between AC and CC transformations should be guided by the specific data structure, as our findings indicate that AC methods like AAC are more advantageous in certain contexts.

### 3.2 Simulation using the GUniFrac package [119]

We utilized the SimulateMSeq function from the GUniFrac package [119] to generate microbiome data simulations, using the human gut metagenome [121] as a reference. The simulation begins by filtering real datasets to remove rare taxa, ensuring that the reference captures essential compositional variations. An empirical Bayes model then estimates the underlying microbial compositions, with Dirichlet hyperparameters derived from observed counts. These compositions are multiplied by a microbial load factor modeled with a log-normal distribution to compute absolute abundances. Covariate and confounder effects are integrated by applying specific coefficients to the absolute abundances, reflecting true biological variability. Sequencing depths are simulated using a negative binomial distribution, adjusting the compositions to produce realistic read counts. This comprehensive approach ensures that the simulated datasets reflect the variability, zero-inflation, and compositional characteristics typical of real microbiome data, making them highly representative of actual scenarios in microbiome analysis [26].

The simulation study was designed with various configurations to represent different scenarios of OTU differential abundance and sequencing depth. We included both “unbalanced” and “balanced” configurations to simulate skewed and evenly distributed differential abundances of OTUs. Different abundance levels were represented by the “rare”, “mix”, and “abundant” modes. We varied the average number of sequences per sample, represented by the values 10, 100, 1,000, and 10,000, and controlled sequencing depth dispersion with values set at 5, 10, and 15. Additionally, we accounted for variability in covariate and confounder effects, with standard deviations set at 0 and 0.5, and controlled the dependence of sequencing depth on the covariate of interest with factors also set at 0 and 0.5. Each parameter combination was subjected to 100 independent simulation runs to ensure statistical robustness and reliability.

To generate the table, we performed simulations using these parameters and calculated the mean power for each transformation. We then conducted linear regression analysis, using the mean power as the dependent variable (y) and the other parameters as independent variables (x). The coefficients from these linear regressions are presented in Table 4.

**Table 4:** Comparison of the effectiveness of various transformations under different conditions using data simulated by SimulateMSeq. The coefficients indicate the impact of diverse data characteristics on the testing power of each method.

Transformation	Intercept	**Table:** Differential Abundance Pattern (unbalanced)	**Table:** Differential OTU Mode (mix)	**Table:** Differential OTU Mode (rare)	**Table:** Average Sequencing Depth	**Table:** Sequencing Depth Dispersion	**Table:** Covariate Effect Variability	**Table:** Confounder Effect Variability	**Table:** Sequencing Depth- Covariate Dependence
ALR	0.2028583	-0.0140753	-0.1176483	-0.2102865	-0.0000032	0.0005273	0.0371649	0.0014363	0.0771814
CLR	0.2360945	-0.0411319	-0.1026250	-0.1859688	-0.0000022	0.0005901	0.0318194	0.0027917	0.1398889
ALTC	0.2051875	-0.0150747	-0.1192861	-0.2128425	-0.0000029	0.0005524	0.0365919	0.0014232	0.0757862
CLTC	0.2373464	-0.0413785	-0.1036927	-0.1877396	-0.0000020	0.0005776	0.0319097	0.0024236	0.1369514
AAC	0.2371161	-0.0476764	-0.1278716	-0.2310723	0.0000043	0.0003289	0.0260472	0.0011916	0.0127752
CAC	0.2450212	-0.0418819	-0.1126927	-0.2181458	0.0000046	0.0003578	0.0197222	0.0020139	0.0513333
APC	0.0243141	-0.0282513	-0.0363470	-0.0629879	-0.0000056	0.0050506	0.0109311	-0.0004762	1.0308448
CPC	0.0280524	-0.0404306	-0.0446615	-0.0753385	0.0000069	0.0027630	0.0167361	-0.0018611	1.1736389
ADGTLC	0.0260923	-0.0114539	-0.0130620	-0.0222931	0.0000006	-0.0000426	0.0075632	-0.0003211	-0.0000809
CDGTLC	0.0334972	-0.0078861	-0.0170749	-0.0307942	0.0000006	-0.0002231	0.0120827	0.0004768	0.0050141
ADGBCC	-0.0553810	-0.0043816	-0.0149387	-0.0160535	-0.0000063	0.0085528	0.0055796	-0.0017481	0.3861693
CDGBCC	-0.0764263	0.0132917	-0.0589896	-0.1026406	0.0000040	0.0112505	0.0053611	-0.0018611	0.8805417
ILR	0.0883522	-0.0173542	-0.0524010	-0.0412708	-0.0000072	0.0003078	0.0156528	0.0046944	0.1855417
Box-Cox in Ratio	0.1872684	-0.0149951	-0.1137799	-0.1932584	-0.0000019	0.0004621	0.0353823	0.0002474	0.0595519
Dual Group Box-Cox in Ratio	0.1961110	-0.0158178	-0.1156872	-0.2028145	-0.0000014	0.0005145	0.0356577	0.0010361	0.0674130

We also conducted a linear regression analysis for false discovery rate (FDR), following the same approach as for power. The coefficients from these FDR regressions are presented in a Supplementary table in Section 9. Interestingly, we observed that the FDR for each transformation is inversely correlated with power, indicating that transformations with higher power tend to have lower FDR.

The intercept values in the table indicate that the CAC transformation exhibits the highest intercept. Additionally, the CLTC and the Centered Log Ratio transformations also show relatively high intercepts compared to other transformations, suggesting their robustness.

An interesting observation is that the coefficient for sequencing depth is positive for both the AAC and CAC transformations. This contrasts with most other transformations, which generally have negative or negligible coefficients for sequencing depth. The positive coefficient implies that, for these transformations, an increase in the average number of sequences per sample is associated with a higher mean power. This may indicate that these transformations are particularly effective at high sequencing depths to improve statistical power.

Furthermore, the coefficient for the dispersion of sequencing depth is relatively smaller for both the AAC and CAC transformations compared to other transformations with similarly high intercepts. This suggests that these transformations are less sensitive to variability in sequencing depth, making them more robust in scenarios with variable sequencing depth.

In summary, the CAC transformation and CLTC transformation stand out due to their high intercepts, making them preferable choices in many practical microbiome data analysis scenarios. The AAC also merits attention for its high intercept and robustness, particularly in its unique positive association with higher sequencing depths and lower sensitivity to sequencing depth variability.

## 4 Evaluation of transformation methods on human gut microbiota data [121]

Research has shown a strong link between the gut microbiome and inflammatory bowel disease (IBD), which includes chronic conditions like Crohn's disease and ulcerative colitis that inflame the gastrointestinal tract. Among recent studies, Mills et al. [121] examines how the gut bacterium Bacteroides vulgatus aggravates colitis, particularly through the role of its proteases in promoting inflammation and disrupting the gut barrier. Through a multi-omics approach combining metagenomics, metaproteomics, and microbiome data, the study identifies a subset of ulcerative colitis (UC) patients with elevated levels of B. vulgatus proteases, which are linked to increased disease severity. In this section, we applied the discussed transformation methods to 16S rRNA sequencing data from this study to compare our proposed methods with existing ones, aiming to demonstrate the enhanced robustness of our methods in differential abundance analyses.

Initially, the data, consisting of 206 samples, was reorganized by consolidating samples from various diagnostic groups into two categories—healthy controls and all other diagnoses. Before applying any preprocessing methods, taxa containing more than 90% zero values were filtered out, reducing the number of taxa from 7019 to 211.

DESeq2 is a popular tool used for analyzing count-based data from RNA sequencing (RNA-seq) experiments [28] and is also frequently applied in microbiome differential abundance analysis. It scales count data by a size factor calculated using the median count ratio across rows and columns, making it incompatible with zero values. To handle zero values in the data, two commonly used preprocessing methods were employed in DESeq2: (1) using the ‘poscounts’ estimator, which handles genes with zero values by calculating a modified geometric mean, specifically the n-th root of the product of the non-zero counts [49], which supported by Van den Berge et al. [122], and (2) replacing zero values with 1.

We applied DESeq2 to the amplicon sequence variant (ASV) count data using the two preprocessing methods for handling zeros described above. The first method resulted in the detection of 6 significant features, while the second method detected 98 significant features. This variation suggests that DESeq2 does not handle zero values very well, producing inconsistent results. The choice of method for handling zeros can lead to significantly different results.

Next, we used Total Sum Scaling (TSS) to scale the count data into relative abundances. For transformations that cannot handle zeros, we replaced zeros with 0.5 prior to performing TSS. We then applied the transformation methods to the relative abundance data and conducted two-sample t-tests to identify significant features. For the AC transformations, which require a reference, we first identified the most non-significant variable using a series of statistical tests (e.g., Wilcoxon test [85]) and used this variable as the reference. The results were then compared with the significant features identified by DESeq2 in both preprocessing methods. The overlap between DESeq2 and our transformation methods is summarized in Table 5.

**Table 5:** Comparison of significant features detected by DESeq2 and various transformation methods using two-sample t-tests. The table lists the number of overlapping significant features, those detected only by DESeq2, and those detected only by the t-test for each transformation method.

Different Preprocessing Methods for DESeq2	Using Modified Geometric Mean			Replace 0 With 1		
Transformation	Overlap	DESeq2y	T Test Only	Overlap	DESeq2y	T Test Only
ALR	0	6	27	14	84	13
CLR	3	3	74	34	64	43
ALTC	0	6	27	14	84	13
CLTC	3	3	74	34	64	43
AAC	0	6	11	6	92	5
CAC	3	3	70	36	62	37
APC	0	6	0	0	98	0
CPC	0	6	11	4	94	7
ADGTLC	0	6	3	2	96	1
CDGTLC	1	5	26	13	85	14
ADGBCC	0	6	0	0	98	0
CDGBCC	0	6	2	0	98	2
ILR	0	6	54	0	98	54
Box-Cox in Ratio	0	6	23	11	87	12
Dual Group Box-Cox in Ratio	0	6	25	0	98	25

From Table 5, different compositional transformations produce notably varied results, illustrating a common phenomenon in microbiome analysis: various differential analysis tools often yield inconsistent outcomes. Notably, the CAC method exhibits the highest overlap with DESeq2 results (3 overlaps using the ‘poscounts’ estimator, and 36 overlaps when replacing zero values with 1), indicating strong concordance. While other transformations such as CLR and CLTC also show high overlaps, the CAC method stands out by having the lowest number of t-test-only significant features in both methods, suggesting a lower false discovery rate (FDR). However, relying solely on overlap may not provide a comprehensive evaluation, given the lack of a true ground truth in real data. We further analyzed the transformed data by calculating the mean and standard deviation (SD) of skewness and kurtosis for each transformation method across the two groups (healthy controls as A and all other diagnoses as B) to assess the normality and distribution characteristics of the transformed data.

The table for skewness and kurtosis was included in our Supplementary materials in Section 10. Although the CAC transformation did not exhibit the best conversion skewness and kurtosis compared to others, such as the CPC, it demonstrates a good balance between conversion and maintaining the signal. This balance is crucial for reliable parametric statistical tests and the detection of significant features in microbiome data analysis.

## 5 Conclusion

When analyzing microbiome data, researchers often debate between two major approaches: count data analysis and compositional data analysis. Despite the argument for considering the compositional nature of microbiome data [18], [10], [100], [70], [97], a significant portion of microbiome data analysis still relies on count data, as seen with differential abundance tools like edgeR [29], LEfSe [30], DESeq2 [28] and ANCOM-BC [27]. Preprocessing microbiome data through count data scaling and compositional data transformation is critical to prepare the data for downstream analyses, helping to mitigate heterogeneity and release constraints. But both count data and compositional data approaches introduce biases and yield inconsistent results on the same data. To address these discrepancies, we have systematically reviewed current transformation techniques for microbiome data analysis and introduced a novel framework that combines proportion conversion with contrast transformations. This innovative approach provides microbiome researchers with a significant direction to enhance data transformation procedures and improve analytical outcomes. Its impact extends beyond immediate research outcomes, shaping the evolution of microbiome data analysis and advancing accurate discoveries in the broader field of microbiome science.

Through extensive simulations using Zero-Inflated Negative Binomial (ZINB) models and the GUniFrac simulation framework, we found that our proposed methods, particularly the Additive Arcsine Contrast (AAC) and Centered Arcsine Contrast (CAC) transformations, consistently outperformed traditional approaches. These methods excel not only because they eliminate the need for biased zero replacement—a common issue in highly zero-inflated datasets such as microbiome data—but also because they demonstrate remarkable stability across various conditions, including varying sequence depths and scenarios with subtle differential abundance signals.

Furthermore, our real data analyses revealed that DESeq2 produced markedly different results depending on the choice of size factor, underscoring the critical importance of the transformation step in differential analysis. This finding highlights that transformation is not merely a procedural necessity but a decisive factor that directly impacts analytical outcomes in real-world applications. While existing tools typically involve multi-step pipelines—including decisions on size factor selection, scaling, transformation, and methods to mitigate the influence of outliers—our proposed transformations, AAC and CAC, provide critical robustness in the initial steps, enhancing the reliability of subsequent analyses, particularly in zero-inflation scenarios. These transformations streamline the analytical process and have the potential to be integrated to enhance the reliability of results across various differential analysis tools.

In this paper, we unify and refine compositional data transformation approaches, developing new methods to manage within-sample compositionality and across-sample variability. Our framework offers a flexible solution for normalizing compositional data, allowing researchers to adapt proportional conversion methods and specific contrast transformations to their unique analytical needs. This adaptability ensures that the data meets the assumptions of common statistical methods, thereby enhancing the accuracy and reliability of subsequent analyses.

Our new framework for robust data transformation is indispensable for unlocking the full quantitative potential of microbiome research. Given its interdisciplinary nature, adopting thoroughly justified, precise, and reliable biostatistical and computational methods will be critical in translating quantitative insights into tangible health benefits. This proposed framework offers a promising direction for future research focused on the development and validation of new transformation techniques, ensuring meaningful and impactful progress in the field.

We encourage researchers to adopt and refine the methods and framework discussed in this review, contributing to the collective effort to improve data analysis in microbiome research. By addressing the limitations and building on the strengths of current techniques, the scientific community can continue to make significant strides in understanding the intricate relationships within microbial ecosystems and their effects on human health.

## 6 Discussion

Our proposed framework, which combines the conversion of proportional data with contrast transformations, presents significant advancements in compositional data analysis. By consolidating existing methods into a structured framework, akin to the periodic table, we systematically organize approaches to clarify relationships among current methods and lay a foundation for developing new methodologies within this structured context. This unified framework not only addresses key challenges but also offers a comprehensive assessment of existing analytical issues and misconceptions in microbial analysis.

A key limitation of our framework is its design specifically for compositional data, where values sum to one. In cases involving count data, methods like TSS are often required to scale counts into relative abundances before application. This transformation may not fully capture the nuances of the original data, potentially reducing its accuracy. A future direction would be adapting the framework to work directly with raw count data, broadening its applicability and enabling more direct microbiome analysis without prior transformations.

Additionally, the contrast transformation employed here is based on differences rather than ratios, emphasizing deviations between variables instead of fold changes. While this approach offers simplicity and interpretability, it may overlook the multiplicative relationships between components in some datasets. The log conversion, a special case in proportional data conversion due to its application of the Quotient Rule, preserves ratio-based relationships. Exploring alternative transformations that preserve ratio-based relationships could improve insights, especially in contexts where relative changes are critical.

Furthermore, while this paper focuses on additive and centered contrasts, other contrast transformations such as pairwise contrasts [92] and pivot contrasts [88], [92] are also commonly employed in compositional data analysis. Nonlinear contrasts like amalgamation contrasts offer additional approaches as well [95], [92]. Future research could explore these transformations within our framework, potentially enhancing flexibility and performance across diverse datasets.

Another aspect with room for future development in the paper is that we evaluated the performance of these transformations solely using the traditional t-test. Our decision to employ fundamental tests, such as the t-test and Wilcoxon rank-sum test, stems from their simplicity, widespread use, and adaptability to more advanced methods like regression. Our goal was to demonstrate that if these basic tests perform well with our proposed transformations, then more sophisticated tests would also be applicable and potentially even more effective. We showed that AAC and CAC outperformed traditional methods in differential abundance testing. These results establish a pathway that makes the proposed CCT framework readily adaptable for the size factor calculation step in developing new differential analysis tools, even for those requiring non-normal distributions.

Additionally, AAC and CAC, as compositional data transformations, can be used beyond differential abundance testing, as demonstrated in our manuscript, and are also suitable for other applications, such as distance-based approaches. For instance, we explored the use of Euclidean distance and found that replacing zeros and using CLR transformation before calculating the Euclidean distance significantly affected the results, with different pseudo-counts leading to substantial variations in the calculated distances. Further details are provided in our Supplementary Section 13. Importantly, unlike traditional methods, AAC and CAC do not require the use of pseudo-counts, making them more robust in handling zero-inflated data. Furthermore, our new framework can also be applied in other areas, such as variable selection [123] (in our Supplementary Section 14) or predictive modeling.

In summary, we provided a precise critique of the unsuitability of compositional data analysis in omics applications, systematically evaluating the widespread but misguided practices that have persisted over time. For microbial analysis, we presented and summarized numerous existing analytical issues and misconceptions in thorough detail, offering a comprehensive assessment and proposing solutions. While this framework addresses key challenges in compositional data analysis, its limitations provide a roadmap for future improvements, particularly in extending its use to raw count data, exploring ratio-preserving transformations, and evaluating its performance using advanced statistical methods.

## Abbreviations

AAC: Additive Arcsine Contrast

AC: Additive Contrast

ADGBCC: Additive Dual Group Box-Cox Contrast

ADGTLC: Additive Dual Group Truncated Logit Contrast

ALR: Additive Log Ratio

ALTC: Additive Logit Contrast

APC: Additive Power Contrast

CAC: Centered Arcsine Contrast

CC: Centered Contrast

CDGBCC: Centered Dual Group Box-Cox Contrast

CDGTLC: Centered Dual Group Truncated Logit Contrast

CCT: Conversion And Contrast Transformation

CLR: Centered Log Ratio

CLTC: Centered Logit Contrast

CPC: Centered Power Contrast

CPM: Counts Per Million

CSS: Cumulative Sum Scaling

DGBC: Dual Group Box-Cox

DGTL: Dual Group Truncated Logit

FDR: False Discovery Rate

GMPR: Geometric Mean Of Pairwise Ratios

HTS: High-Throughput Sequencing

ILR: Isometric Log Ratio

OTU: Operational Taxonomic Unit

TMM: Trimmed Mean Of M-Values

TSS: Total Sum Scaling

UQ: Upper Quartile

ZINB: Zero Inflated Negative Binomial

## Code and data availability statement

The code used in this study are openly available and can be accessed through the following GitHub repository: https://github.com/bioscinema/Microbiome_Transformation.

For the IBD data used in the manuscript in Section 4, details are also provided in the GitHub repository. The Hadza microbiome dataset used in the supplementary material is available upon request.

## CRediT authorship contribution statement

**Yiqian Zhang:** Writing – review & editing, Writing – original draft, Visualization, Software, Methodology, Formal analysis, Conceptualization. **Jonas Schluter:** Writing – review & editing. **Lijun Zhang:** Writing – review & editing. **Xuan Cao:** Writing – review & editing. **Robert R. Jenq:** Writing – review & editing. **Hao Feng:** Writing – review & editing. **Jonathan Haines:** Writing – review & editing. **Liangliang Zhang:** Writing – review & editing, Visualization, Supervision, Resources, Project administration, Methodology, Funding acquisition, Conceptualization.

## Declaration of Competing Interest

The authors declare that they have no known competing financial interests or personal relationships that could have appeared to influence the work reported in this paper.

## Appendix A Supplementary material

MMC This supplementary document provides additional information to support the main manuscript titled “Review and revamp of compositional data transformation: a new framework combining proportion conversion and contrast transformation”. It includes supplementary figures, tables, and detailed methods. MMC
