# Supplementary material for: Review and revamp of compositional data transformation: A new framework combining proportion conversion and contrast transformation
Source: Comput Struct Biotechnol J. 2024 Nov 8;23:4088–107. doi: 10.1016/j.csbj.2024.11.003 (PMC11609487; doi:10.1016/j.csbj.2024.11.003)
Supplement: MMC — This supplementary document provides additional information to support the main manuscript titled “Review and revamp of compositional data transformation: a new framework combining proportion conversion and contrast transformation”. It includes supplementary figures, tables, and detailed methods. [file mmc1.pdf]

Supplementary Material of "Review and Revamp of Compositional Data Transformation: A New Framework Combining Proportion Conversion and Contrast Transformation"

Yiqian Zhang, Jonas Schluter, Lijun Zhang, Xuan Cao, Robert R Jenq, Hao Feng, Jonathan Haines, Liangliang Zhang*

This supplementary document provides additional information to support the main manuscript titled "Review and Revamp of Compositional Data Transformation: A New Framework Combining Proportion Conversion and Contrast Transformation". It includes supplementary figures, tables, and detailed methods.

1 Arcsine Normal Distribution

The arcsine normal distribution is derived from the arcsine conversion of a variable that is normally distributed. The arcsine conversion is defined as:

$$y = \frac{2}{\pi} \arcsin(\sqrt{x})$$

To derive the arcsine normal distribution, we start by considering the inverse transformation:

$$x = \sin^2\left(\frac{\pi y}{2}\right)$$

The derivative of the inverse function with respect to y is:

$$\frac{dx}{dy} = \pi \sin\left(\frac{\pi y}{2}\right) \cos\left(\frac{\pi y}{2}\right)$$

Therefore,

$$\frac{dy}{dx} = \frac{1}{\frac{dx}{dy}} = \frac{1}{\pi \sin\left(\frac{\pi y}{2}\right) \cos\left(\frac{\pi y}{2}\right)} = \frac{1}{\pi\sqrt{x}\sqrt{1-x}}$$

Given that y follows a normal distribution with mean μ and standard deviation σ, the probability density function (PDF) of y is:

$$f_y(y) = \frac{1}{\sigma\sqrt{2\pi}} \exp\left(-\frac{(y-\mu)^2}{2\sigma^2}\right)$$

Using the change of variables formula, the PDF of x can be derived as follows:

$$f_x(x) = f_y(g^{-1}(x)) \left|\frac{dy}{dx}\right|$$

Substituting the inverse transformation $y = \frac{2}{\pi}\arcsin(\sqrt{x})$ and the derivative $\frac{dy}{dx} = \frac{1}{\pi\sqrt{x}\sqrt{1-x}}$:

$$f_x(x) = \frac{1}{\sigma\sqrt{2\pi}} \exp\left(-\frac{\left(\frac{2}{\pi}\arcsin(\sqrt{x}) - \mu\right)^2}{2\sigma^2}\right) \left|\frac{1}{\pi\sqrt{x}\sqrt{1-x}}\right|$$

Simplifying the expression, we get:

$$f_x(x) = \frac{1}{\sigma\sqrt{2\pi}} \exp\left(-\frac{\left(\frac{2}{\pi}\arcsin(\sqrt{x}) - \mu\right)^2}{2\sigma^2}\right) \cdot \frac{1}{\pi\sqrt{x}\sqrt{1-x}}$$

*Corresponding author: Liangliang Zhang, Ph.D, lxz716@case.edu

Therefore, the arcsine normal distribution is given by:

$$f_x(x) = \frac{1}{\sigma\sqrt{2\pi}} \exp\left(-\frac{\left(\frac{2}{\pi}\arcsin(\sqrt{x}) - \mu\right)^2}{2\sigma^2}\right) \cdot \frac{1}{\pi\sqrt{x}\sqrt{1-x}}$$

Thus, if a random variable x follows the arcsine normal distribution, then after the arcsine conversion $y = \frac{2}{\pi}\arcsin(\sqrt{x})$, it will follow a normal distribution.

Different from the log-normal [1] and logit-normal [2] distributions, where y approaches 0 as x approaches 0, the arcsine normal distribution has the property that y approaches infinity as x approaches 0. This characteristic can be advantageous in certain situations where the data behavior near zero needs to be modeled with a more pronounced asymptote. For example, in microbiome research, where very small values of x (e.g., near-zero proportions of specific microbial species) are expected to have a significant impact or require special emphasis, the arcsine normal distribution provides a more appropriate fit by accentuating the tails and giving more weight to small values. This can lead to better modeling and interpretation of extreme low values compared to the log-normal and logit-normal distributions, improving the accuracy of analyses in studies involving rare microbial taxa.

2 Impact of Reference Choice on ALR Transformation

We have created several figures to demonstrate that choosing different references for the Additive Log-Ratio (ALR) transformation can cause significant changes in the differential abundance test. These figures are based on real data from tumor microbiome studies. The figures are as follows:
- **Figure S1(a)**: Replace 0 with 0.5 in count data, without removing outliers.
- **Figure S1(b)**: Replace 0 with 0.5 in count data, with removing outliers.
- **Figure S1(c)**: Replace 0 with 1 in count data, without removing outliers.
- **Figure S1(d)**: Replace 0 with 1 in count data, with removing outliers.

We performed two group t-tests on pancreatic tumor microbiome data [3] to assess the effect of different reference selections on the additive log-ratio (ALR) transformation for differential analysis. In the resulting figure, both the x-axis and y-axis represent variable positions within the dataset. The blue diagonal line indicates the chosen reference, progressing from the first to the last position across the data. The red dots along the y-axis represent the variables that were found to be significant. In other words, the x-axis corresponds to the variables selected as references, and examining the plot vertically shows which variables are deemed significant for each specific reference. This approach highlights how the choice of reference can impact the identification of significant variables in the analysis.

All four figures are displayed in a 2x2 grid format below for comparison.

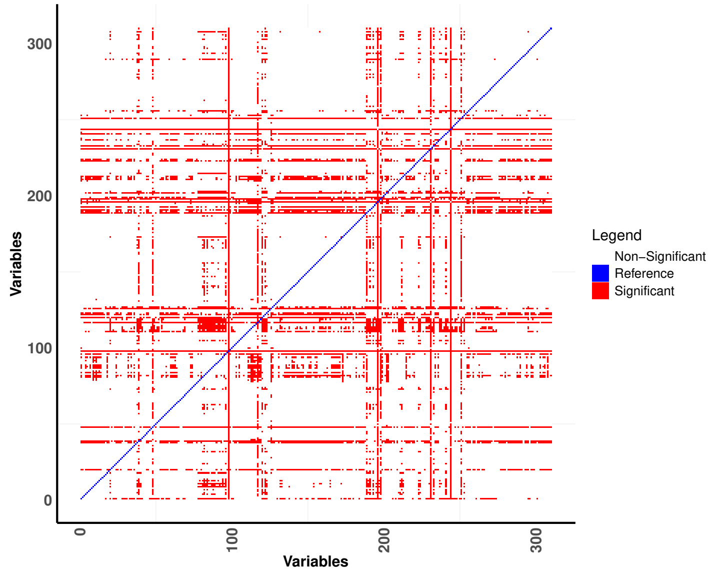

(a)

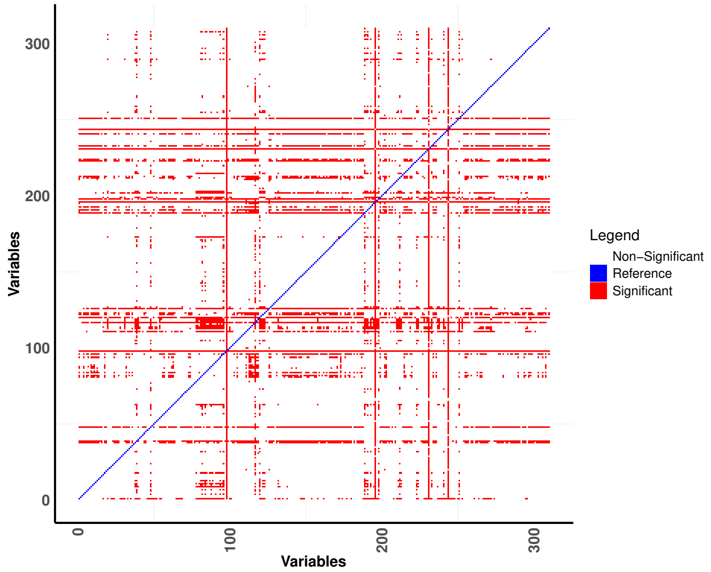

(b)

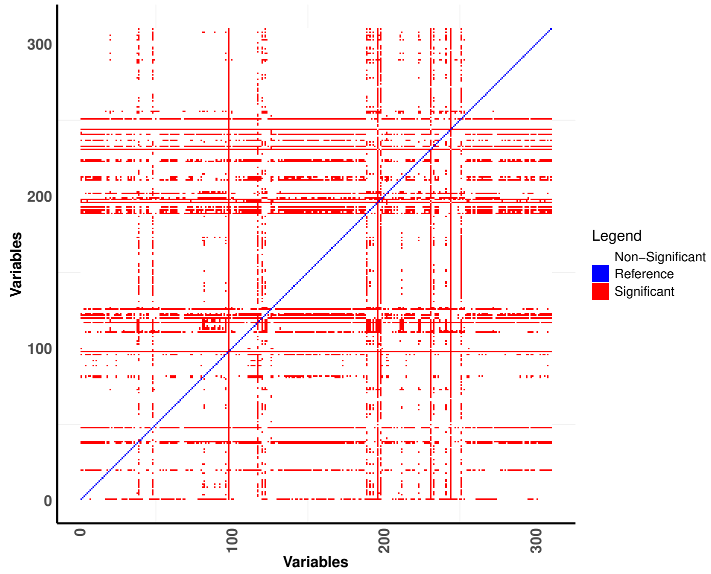

(c)

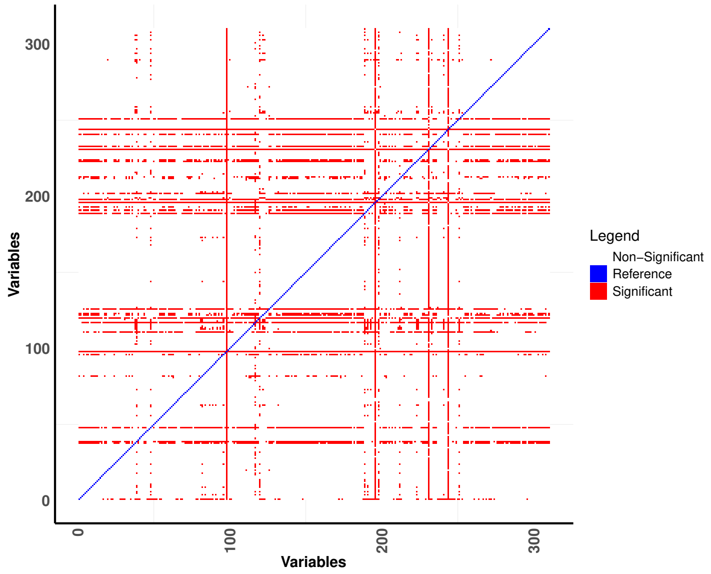

(d)

Figure S1: Comparison of ALR transformation results with different reference choices and outlier treatments.

From the perspective of outliers, removing outliers reduces the noise points in the graph, which decreases the possibility of false discoveries. Additionally, we found that using different values to replace zeros in the data also significantly changed the results. Some references caused more than 80% significance rates for all other variables. Upon further investigation, we found that these references were themselves strongly significant in terms of differential abundance. This indicates that choosing significant variables as references can greatly influence the results of other variables.

Based on these observations, we recommend that when choosing a reference for the ALR transformation, it is crucial to select a reference that is not significant. Additionally, removing outliers should be considered as it can help reduce noise and potential false discoveries. The choice of value to replace zeros also requires careful attention to ensure the robustness of the results.

Algorithm 1 Outlier Removal Method

```
function REMOVE OUTLIERS(DataFrame, Group Column)
    For each numeric column in the DataFrame:
        Filter out zero values.
        Compute the logarithm of the non-zero values.
        Calculate the first quartile (Q1) and third quartile (Q3).
        Determine the interquartile range (IQR) as Q3 - Q1.
        Compute the lower bound as Q1 - 1.5 * IQR.
        Compute the upper bound as Q3 + 1.5 * IQR.
        Retain values within the lower and upper bounds.
        Set values outside the bounds to NA.
    Apply this process within each group defined by the Group Column.
    Return the cleaned DataFrame with outliers removed.
end function
```

3 New Proposed Proportional Data Conversion

3.1 Truncated Logit Conversion

Traditional logit conversion encounters issues at the boundary values of 0 and 1. To overcome this, we propose a novel truncated logit conversion:

$$y_i = \log\left(\frac{p_i + \phi}{1 - p_i + \varphi}\right), \quad \phi \geq 0, \varphi \geq 0,$$

where p_i is proportion data, ϕ represents the truncation parameter at 0, and φ represents the truncation parameter at 1.

The objective is to transform the proportion data into a normal-like distribution, allowing the converted data to be modeled by a normal density function. The density function for this conversion is:

$$\pi(\phi, \varphi, \mu, \sigma^2) = \prod_{i=1}^{n} (2\pi\sigma^2)^{-\frac{1}{2}} \exp\left[-\frac{1}{2\sigma^2}\left(\log\left(\frac{p_i + \phi}{1 - p_i + \varphi}\right) - \mu\right)^2\right] \times \frac{\phi + \varphi + 1}{(p_i + \phi)(\varphi + 1 - p_i)},$$

where μ is the mean and σ^2 is the variance of the converted normal distribution. This density is well-defined for boundary values of p (i.e., 0 and 1) when $\phi \geq 0$ and $\varphi \geq 0$.

We derive the log-likelihood function as:

$$L(\phi, \varphi, \mu, \sigma^2) = -\frac{n}{2}\log(2\pi\sigma^2) - \frac{1}{2\sigma^2}\sum_{i=1}^{n}\left(\log\left(\frac{p_i + \phi}{1 - p_i + \varphi}\right) - \mu\right)^2 + n\log(\phi + \varphi + 1) - \sum_{i=1}^{n}\log\left((p_i + \phi)(\varphi + 1 - p_i)\right),$$

subject to the constraints $\phi \geq 0$ and $\varphi \geq 0$.

To simplify the estimation, we first calculate the maximum likelihood estimates (MLE) of μ and σ^2 using the profile likelihood method. The score functions for μ and σ^2 are:

$$\frac{dL}{d\mu} = -\frac{1}{\sigma^2}\sum_{i=1}^{n}\left(\mu - \log\left(\frac{p_i + \phi}{1 - p_i + \varphi}\right)\right) = 0,$$

$$\frac{dL}{d\sigma^2} = -\frac{n}{2}\frac{1}{\sigma^2} + \frac{1}{2(\sigma^2)^2}\sum_{i=1}^{n}\left(\log\left(\frac{p_i + \phi}{1 - p_i + \varphi}\right) - \mu\right)^2 = 0.$$

By solving these equations, we obtain the MLEs:

$$\hat{\mu} = \frac{1}{n}\sum_{i=1}^{n}\log\left(\frac{p_i + \phi}{1 - p_i + \varphi}\right),$$

$$\hat{\sigma}^2 = \frac{1}{n}\sum_{i=1}^{n}(y_i - \hat{\mu})^2,$$

where $y_i = \log\left(\frac{p_i+\phi}{1-p_i+\varphi}\right)$ and $\bar{y} = \hat{\mu}$.

Substituting $\hat{\mu}$ and $\hat{\sigma}$ back into the log-likelihood function, we obtain:

$$L(\phi, \varphi) = -\frac{n}{2}\log\left(2\pi\frac{1}{n}\sum_{i=1}^{n}(y_i - \bar{y})^2\right) - \frac{n}{2} + n\log(\phi + \varphi + 1) - \sum_{i=1}^{n}\log\left((p_i + \phi)(\varphi + 1 - p_i)\right),$$

To identify the optimal parameters, we maximize this log-likelihood function. The optimal values of ϕ and φ are those that maximize the log-likelihood, ensuring the converted data is as close to a normal distribution as possible, facilitating more accurate statistical analysis.

3.2 Dual Group Truncated Logit Conversion

Extending the truncated logit conversion, we propose a model to handle proportion data from two distinct groups. This model is designed to analyze differential effects across groups while maintaining common truncation parameters, ϕ and φ, ensuring methodological consistency and robust comparative analysis.

Given two groups of data, A and B, with proportions p_{Ai} and p_{Bi} respectively, we extend the truncated logit conversion as follows:

$$y_{Ai} = \log\left(\frac{p_{Ai}+\phi}{1-p_{Ai}+\varphi}\right), \quad y_{Bi} = \log\left(\frac{p_{Bi}+\phi}{1-p_{Bi}+\varphi}\right),$$

where $\phi \geq 0$ and $\varphi \geq 0$ are the shared truncation parameters at 0 and 1 respectively.

The joint likelihood for both groups, assuming independence between them, is formulated as the product of their individual likelihoods:

$$\begin{aligned}\pi(\phi,\varphi,\mu_A,\sigma_A^2,\mu_B,\sigma_B^2) = &\left[\prod_{i=1}^{n_A}(2\pi\sigma_A^2)^{-\frac{1}{2}}\exp\left(-\frac{1}{2\sigma_A^2}(y_{Ai}-\mu_A)^2\right)\cdot\frac{\phi+\varphi+1}{(p_{Ai}+\phi)(\varphi+1-p_{Ai})}\right]\\ &\times\left[\prod_{i=1}^{n_B}(2\pi\sigma_B^2)^{-\frac{1}{2}}\exp\left(-\frac{1}{2\sigma_B^2}(y_{Bi}-\mu_B)^2\right)\cdot\frac{\phi+\varphi+1}{(p_{Bi}+\phi)(\varphi+1-p_{Bi})}\right],\end{aligned}$$

where μ_A, σ_A^2, μ_B, and σ_B^2 are the means and variances of the converted normal distributions for groups A and B respectively.

Given the joint likelihood function for groups A and B, the log likelihood can be expressed as:

$$\begin{aligned}&L(\phi,\varphi,\mu_A,\sigma_A^2,\mu_B,\sigma_B^2) =\\ &-\frac{n_A}{2}\log(2\pi\sigma_A^2)-\frac{1}{2\sigma_A^2}\sum_{i=1}^{n_A}(y_{Ai}-\mu_A)^2+n_A\log(\phi+\varphi+1)-\sum_{i=1}^{n_A}\log((p_{Ai}+\phi)(\varphi+1-p_{Ai}))\\ &-\frac{n_B}{2}\log(2\pi\sigma_B^2)-\frac{1}{2\sigma_B^2}\sum_{i=1}^{n_B}(y_{Bi}-\mu_B)^2+n_B\log(\phi+\varphi+1)-\sum_{i=1}^{n_B}\log((p_{Bi}+\phi)(\varphi+1-p_{Bi})),\end{aligned}$$

To find the maximum likelihood estimates for μ_A, σ_A^2, μ_B, and σ_B^2, we take the partial derivatives of the log likelihood function with respect to these parameters and set them to zero.

For group A, the score functions are:

$$\begin{aligned}\frac{\partial L}{\partial\mu_A} &= -\frac{1}{\sigma_A^2}\sum_{i=1}^{n_A}(y_{Ai}-\mu_A) = 0,\\ \frac{\partial L}{\partial\sigma_A^2} &= -\frac{n_A}{2\sigma_A^2}+\frac{1}{2(\sigma_A^2)^2}\sum_{i=1}^{n_A}(y_{Ai}-\mu_A)^2 = 0.\end{aligned}$$

Solving these for μ_A and σ_A^2 gives:

$$\begin{aligned}\hat{\mu}_A &= \frac{1}{n_A}\sum_{i=1}^{n_A}y_{Ai},\\ \hat{\sigma}_A^2 &= \frac{1}{n_A}\sum_{i=1}^{n_A}(y_{Ai}-\hat{\mu}_A)^2.\end{aligned}$$

Similarly, for group B, the maximum likelihood estimates are obtained by:

$$\begin{aligned}\hat{\mu}_B &= \frac{1}{n_B}\sum_{i=1}^{n_B}y_{Bi},\\ \hat{\sigma}_B^2 &= \frac{1}{n_B}\sum_{i=1}^{n_B}(y_{Bi}-\hat{\mu}_B)^2.\end{aligned}$$

where $y_{Ai} = \log\left(\frac{p_{Ai}+\phi}{1-p_{Ai}+\varphi}\right)$, $\bar{y}_A = \hat{\mu}_A$, $y_{Bi} = \log\left(\frac{p_{Bi}+\phi}{1-p_{Bi}+\varphi}\right)$ and $\bar{y}_B = \hat{\mu}_B$.

After plugging $\hat{\mu}_A$, $\hat{\sigma}_A$, $\hat{\mu}_B$, and $\hat{\sigma}_B$ back into the log likelihood for our Dual-group Logit Conversion, we obtain:

$$\begin{aligned}L(\phi,\varphi) = &-\frac{n_A}{2}\log(2\pi\frac{1}{n_A}\sum_{i=1}^{n_A}(y_{Ai}-\bar{y}_A)^2)-\frac{n_A}{2}+n_A\log(\phi+\varphi+1)-\sum_{i=1}^{n_A}\log((p_{Ai}+\phi)(\varphi+1-p_{Ai}))\\ &-\frac{n_B}{2}\log(2\pi\frac{1}{n_B}\sum_{i=1}^{n_B}(y_{Bi}-\bar{y}_B)^2)-\frac{n_B}{2}+n_B\log(\phi+\varphi+1)-\sum_{i=1}^{n_B}\log((p_{Bi}+\phi)(\varphi+1-p_{Bi})),\end{aligned}$$

As with the truncated logit conversion, we maximize this log-likelihood function to choose the best parameters for the Dual-Group Logit conversion. By finding the values of ϕ and φ that maximize the log-likelihood, we ensure that the transformed data conforms as closely as possible to a normal distribution, facilitating more accurate and reliable statistical analysis. This approach is particularly advantageous for comparative studies requiring methodological consistency between groups, as it uses common truncation parameters, ϕ and φ, to facilitate robust and reliable comparisons. By effectively handling boundary values and applying consistent conversion parameters across groups, this method enhances the accuracy of differential effect analysis, improves the power and sensitivity of statistical tests, and ensures that observed differences are due to actual variations in the data rather than conversion artifacts.

3.3 Dual Group Boxcox Conversion

Based on the traditional Boxcox Conversion, We propose the Dual-Group Box-Cox Conversion as follows:

$$y_{A_i} = \begin{cases} \frac{(x_{A_i})^{\lambda}-1}{\lambda} & \text{if } \lambda \neq 0 \\ \log(x_{A_i}) & \text{if } \lambda = 0 \end{cases}, \quad y_{B_i} = \begin{cases} \frac{(x_{B_i})^{\lambda}-1}{\lambda} & \text{if } \lambda \neq 0 \\ \log(x_{B_i}) & \text{if } \lambda = 0 \end{cases}$$

The likelihood function for the Dual-Group Box-Cox Conversion, considering the parameters λ, μ_A, σ_A^2, μ_B, and σ_B^2, can be formulated as follows:

$$\begin{aligned} \pi(\lambda, \mu_A, \sigma_A^2, \mu_B, \sigma_B^2) = & \left[\prod_{i=1}^{n_A} (2\pi\sigma_A^2)^{-\frac{1}{2}} \exp\left(-\frac{1}{2\sigma_A^2} (y_{A_i} - \mu_A)^2 \right) (x_{A_i})^{\lambda-1} \right] \\ & \times \left[\prod_{i=1}^{n_B} (2\pi\sigma_B^2)^{-\frac{1}{2}} \exp\left(-\frac{1}{2\sigma_B^2} (y_{B_i} - \mu_B)^2 \right) (x_{B_i})^{\lambda-1} \right] \end{aligned}$$

The log-likelihood for this conversion is given by:

$$\begin{aligned} L(\lambda, \mu_A, \sigma_A^2, \mu_B, \sigma_B^2) = & -\frac{n_A}{2} \log(2\pi\sigma_A^2) - \frac{1}{2\sigma_A^2} \sum_{i=1}^{n_A} (y_{A_i} - \mu_A)^2 + (\lambda - 1) \sum_{i=1}^{n_A} \log(x_{A_i}) \\ & - \frac{n_B}{2} \log(2\pi\sigma_B^2) - \frac{1}{2\sigma_B^2} \sum_{i=1}^{n_B} (y_{B_i} - \mu_B)^2 + (\lambda - 1) \sum_{i=1}^{n_B} \log(x_{B_i}) \end{aligned}$$

For group A, the score functions are:

$$\begin{aligned} \frac{\partial L}{\partial \mu_A} &= -\frac{1}{\sigma_A^2} \sum_{i=1}^{n_A} (y_{A_i} - \mu_A) = 0, \\ \frac{\partial L}{\partial \sigma_A^2} &= -\frac{n_A}{2\sigma_A^2} + \frac{1}{2(\sigma_A^2)^2} \sum_{i=1}^{n_A} (y_{A_i} - \mu_A)^2 = 0 \end{aligned}$$

Solving these for μ_A and σ_A^2 gives:

$$\begin{aligned} \hat{\mu}_A &= \frac{1}{n_A} \sum_{i=1}^{n_A} y_{A_i}, \\ \hat{\sigma}_A^2 &= \frac{1}{n_A} \sum_{i=1}^{n_A} (y_{A_i} - \hat{\mu}_A)^2 \end{aligned}$$

Similarly, for group B, the maximum likelihood estimates are obtained by:

$$\begin{aligned} \hat{\mu}_B &= \frac{1}{n_B} \sum_{i=1}^{n_B} y_{B_i}, \\ \hat{\sigma}_B^2 &= \frac{1}{n_B} \sum_{i=1}^{n_B} (y_{B_i} - \hat{\mu}_B)^2 \end{aligned}$$

After plugging in $\hat{\mu}_A$, $\hat{\sigma}_A^2$, $\hat{\mu}_B$, and $\hat{\sigma}_B^2$ back into the log-likelihood for our Dual-Group Box-Cox Conversion, we get:

$$\begin{aligned} L(\lambda) = & -\frac{n_A}{2} \log\left(2\pi \frac{1}{n_A} \sum_{i=1}^{n_A} (y_{A_i} - \bar{y}_A)^2 \right) - \frac{n_A}{2} + (\lambda - 1) \sum_{i=1}^{n_A} \log(x_{A_i}) \\ & - \frac{n_B}{2} \log\left(2\pi \frac{1}{n_B} \sum_{i=1}^{n_B} (y_{B_i} - \bar{y}_B)^2 \right) - \frac{n_B}{2} + (\lambda - 1) \sum_{i=1}^{n_B} \log(x_{B_i}) \end{aligned}$$

Then we maximize the log-likelihood to obtain the parameter λ.

4 Algorithm for Zero Inflated Beta Regression Simulation

We simulate data using a zero-inflated beta regression model with the following parameters:

- n: total number of observations
- p: number of variables
- β: coefficient for significant variables
- ν: dispersion parameter
- x: covariates for groups A and B
- β_0: intercept
- q: probability of zero inflation

The simulation is conducted with 100 samples and 50 variables. The specific parameters are as follows: the sample size is $n = 100$, and the number of variables is $p = 50$. The significant coefficient values are $\beta_{\text{significant}} = -0.7$ and $\beta_{\text{significant}} = -0.5$, while the non-significant coefficient is $\eta_{\text{not significant}} = 0$. The dispersion parameter is $\nu = 5$. The covariates are $x = 1$ and 2, and the intercept is $\beta = -2$. Zero-inflation probabilities are $q = 0\%, 30\%, 50\%, 70\%$. These parameters are chosen to simulate zero-inflated beta-distributed data for two groups, A and B. The significant variables are influenced by the covariate x and the coefficient $\eta_{\text{significant}}$, while the non-significant variables are influenced by x and $\eta_{\text{not significant}}$. Zero-inflation is applied to both groups based on the specified probability q.

We use the algorithm described in Algorithm 2 to simulate the zero-inflated beta-distributed data.

Algorithm 2 Simulate Data by Zero-Inflated Beta Regression

Input: $n, p, \beta, \nu, x, \beta_0, q$
Initialize: Data frame to store results
for each variable j **do**
    Generate significant variables:
    for each observation i **do**
        $\mu_i = \frac{\exp(\beta \cdot x_i + \beta_0)}{1+\exp(\beta \cdot x_i + \beta_0)}$
        $y_i \sim \text{Beta}(\mu_i \cdot \nu, (1 - \mu_i) \cdot \nu)$
        Apply zero-inflation: $y_i = y_i \cdot \mathbb{I}(\text{Bernoulli}(1 - q))$
    end for
    Store y_i in the data frame
    Generate non-significant variables:
    for each observation i **do**
        $\mu_i = \frac{\exp(0 \cdot x_i + \beta_0)}{1+\exp(0 \cdot x_i + \beta_0)}$
        $y_i \sim \text{Beta}(\mu_i \cdot \nu, (1 - \mu_i) \cdot \nu)$
        Apply zero-inflation: $y_i = y_i \cdot \mathbb{I}(\text{Bernoulli}(1 - q))$
    end for
    Store y_i in the data frame
end for
Output: Simulated data frame with zero-inflated beta-distributed data

---

5 Analysis of Skewness and Kurtosis for Various Proportional Data Conversion

Table S1: Summary of Skewness and Kurtosis for Various Conversions

η	Conversion	Category	Group	Percent of 0: 0% Mean	0% SD	30% Mean	30% SD	50% Mean	50% SD	70% Mean	70% SD
-0.5	original	skewness	A	1.7473	0.0667	2.1513	0.0792	2.5851	0.0947	3.3773	0.1278
		skewness	B	2.0308	0.0798	2.4321	0.0933	2.8949	0.1028	3.6397	0.1261
		kurtosis	A	6.0844	0.3687	7.8750	0.5044	10.0867	0.6625	15.2163	1.0649
		kurtosis	B	7.6009	0.5283	9.5852	0.6766	12.3014	0.8180	17.3738	1.1014
	Log Conversion	skewness	A	-1.1427	0.0496	-0.4106	0.0353	0.2971	0.0417	1.1548	0.0518
		skewness	B	-0.9006	0.0495	-0.2160	0.0402	0.4533	0.0402	1.2909	0.0550
		kurtosis	A	4.2445	0.2022	1.5859	0.0467	1.3770	0.0359	2.6710	0.1403
		kurtosis	B	3.4987	0.1905	1.5757	0.0429	1.5902	0.0478	3.1395	0.1880
	Logit Conversion	skewness	A	-0.9659	0.0493	-0.3728	0.0343	0.3210	0.0416	1.1741	0.0520
		skewness	B	-0.7546	0.0480	-0.1816	0.0393	0.4761	0.0399	1.3099	0.0550
		kurtosis	A	3.9376	0.1710	1.5987	0.0447	1.4215	0.0383	2.7452	0.1441
		kurtosis	B	3.2734	0.1609	1.5977	0.0413	1.6400	0.0495	3.2192	0.1911
	Arcsine Conversion	skewness	A	0.8375	0.0434	1.0962	0.0460	1.5277	0.0597	2.3044	0.0862
		skewness	B	1.0343	0.0463	1.3272	0.0538	1.7763	0.0668	2.5520	0.0938
		kurtosis	A	3.2445	0.1394	3.6135	0.1813	4.7418	0.2632	8.0420	0.5441
		kurtosis	B	3.7797	0.1937	4.4419	0.2502	5.9322	0.3755	9.6468	0.6893
	Boxcox Conversion	skewness	A	-0.2112	0.0280	-0.2569	0.0264	0.1983	0.0372	0.9769	0.0495
		skewness	B	-0.0359	0.0252	-0.0630	0.0318	0.3343	0.0353	1.0627	0.0522
		kurtosis	A	2.3557	0.0391	1.5332	0.0311	1.2308	0.0223	2.1081	0.1097
		kurtosis	B	2.1653	0.0384	1.5345	0.0288	1.3586	0.0297	2.3279	0.1347
	Tangent Conversion	skewness	A	-5.1333	0.1635	-0.8287	0.0454	0.0430	0.0417	0.9474	0.0500
		skewness	B	-3.9052	0.1472	-0.6854	0.0518	0.1376	0.0420	1.0180	0.0536
		kurtosis	A	30.3983	1.6355	1.8215	0.0851	1.0936	0.0216	2.0336	0.1079
		kurtosis	B	20.5603	1.2714	1.6422	0.0841	1.1320	0.0253	2.2037	0.1331
	Dual group Logit Conversion	skewness	A	-0.7161	0.0327	-0.3728	0.0343	0.3210	0.0416	1.1738	0.0520
		skewness	B	-0.5063	0.0310	-0.1816	0.0393	0.4761	0.0399	1.3097	0.0550
		kurtosis	A	3.1335	0.0830	1.5987	0.0447	1.4215	0.0383	2.7437	0.1437
		kurtosis	B	2.4859	0.0554	1.5977	0.0413	1.6400	0.0495	3.2175	0.1905
	Dual Group Boxcox Conversion	skewness	A	-0.2381	0.0219	-0.2509	0.0256	0.1977	0.0369	0.9769	0.0494
		skewness	B	-0.0610	0.0197	-0.0563	0.0291	0.3340	0.0362	1.0627	0.0522
		kurtosis	A	2.3600	0.0387	1.5293	0.0314	1.2296	0.0213	2.1078	0.1095
		kurtosis	B	2.1471	0.0350	1.5348	0.0276	1.3586	0.0301	2.3283	0.1348
-0.7	Original Data	skewness	A	1.8683	0.0762	2.2494	0.0817	2.7290	0.0983	3.4711	0.1312
		skewness	B	2.2616	0.0955	2.6864	0.1132	3.1195	0.1125	3.8880	0.1234
		kurtosis	A	6.6727	0.4793	8.4334	0.5480	11.0470	0.7266	15.9781	1.1351
		kurtosis	B	9.0225	0.6841	11.3695	0.9233	14.0046	0.9568	19.5683	1.1268
	Log Conversion	skewness	A	-1.0520	0.0590	-0.3414	0.0371	0.3527	0.0374	1.1944	0.0581
		skewness	B	-0.6678	0.0448	-0.0337	0.0431	0.6175	0.0442	1.4764	0.0623
		kurtosis	A	3.9064	0.2390	1.5731	0.0556	1.4440	0.0377	2.8000	0.1684
		kurtosis	B	3.1783	0.1967	1.6915	0.0428	1.9223	0.0776	3.8743	0.2568
	Logit Conversion	skewness	A	-0.8905	0.0570	-0.3048	0.0360	0.3764	0.0374	1.2136	0.0582
		skewness	B	-0.5343	0.0433	-0.0014	0.0424	0.6395	0.0439	1.4950	0.0627
		kurtosis	A	3.6447	0.2032	1.5891	0.0526	1.4911	0.0400	2.8757	0.1726
		kurtosis	B	2.9753	0.1637	1.7198	0.0418	1.9779	0.0798	3.9601	0.2619
	Arcsine Conversion	skewness	A	0.9219	0.0447	1.1785	0.0474	1.6363	0.0630	2.3899	0.0937
		skewness	B	1.2199	0.0585	1.5434	0.0637	1.9993	0.0706	2.8085	0.0954
		kurtosis	A	3.4474	0.1669	3.8787	0.1903	5.2304	0.3166	8.5817	0.6199
		kurtosis	B	4.4287	0.2867	5.4288	0.3676	7.1731	0.4655	11.5084	0.7554
	Boxcox Conversion	skewness	A	-0.2625	0.0286	-0.2441	0.0280	0.2202	0.0351	0.9920	0.0530
		skewness	B	0.0458	0.0277	0.0605	0.0317	0.4551	0.0403	1.1964	0.0557
		kurtosis	A	2.3434	0.0467	1.5046	0.0329	1.2367	0.0220	2.1443	0.1212
		kurtosis	B	2.0794	0.0354	1.5751	0.0290	1.5177	0.0488	2.7090	0.1659
	Tangent Conversion	skewness	A	-4.6411	0.1963	-0.7896	0.0505	0.0695	0.0404	0.9636	0.0540
		skewness	B	-3.3055	0.1251	-0.5079	0.0473	0.2667	0.0457	1.1512	0.0567
		kurtosis	A	25.9057	1.8840	1.7724	0.1093	1.1007	0.0212	2.0736	0.1205
		kurtosis	B	18.0105	1.2224	1.5271	0.0752	1.2436	0.0403	2.5665	0.1629
	Dual Group Logit Conversion	skewness	A	-0.6488	0.0324	-0.3048	0.0360	0.3764	0.0374	1.2128	0.0579
		skewness	B	-0.2963	0.0296	-0.0014	0.0424	0.6395	0.0439	1.4939	0.0625
		kurtosis	A	2.8675	0.0761	1.5891	0.0526	1.4911	0.0400	2.8713	0.1704
		kurtosis	B	2.2217	0.0433	1.7198	0.0418	1.9779	0.0798	3.9518	0.2595
	Dual Group Boxcox Conversion	skewness	A	-0.2975	0.0245	-0.2411	0.0269	0.2201	0.0348	0.9921	0.0529
		skewness	B	0.0189	0.0208	0.0643	0.0308	0.4558	0.0401	1.1969	0.0559
		kurtosis	A	2.3675	0.0469	1.5016	0.0329	1.2361	0.0220	2.1442	0.1209
		kurtosis	B	2.0501	0.0331	1.5739	0.0270	1.5203	0.0481	2.7121	0.1668

Table S2: Summary of Skewness and Kurtosis for Data and After Each Conversion. Lower skewness (closer to 0) and kurtosis (closer to 3) indicate better normality and symmetry in the data distribution.

The table summarizing skewness and kurtosis for various proportional data conversions provides several important insights into their conversion effectiveness. Notably, the dual group logit conversion exhibits better conversion power than the standard logit conversion, as evidenced by lower skewness and kurtosis values across different percentages of zeros. Similarly, the dual group Boxcox conversion generally demonstrates superior conversion power compared to traditional Boxcox conversion, achieving a more balanced distribution between the two groups. This improved balance is crucial for accurate statistical analysis of proportional data.

Despite the arcsine normalized data not showing very good skewness and kurtosis values across all conversions, especially for high zero-inflation data, this is understandable. The arcsine conversion effectively handles zero values, but since arcsine(0) remains 0, the zero values do not change position, which impacts the skewness and kurtosis metrics. This limitation should be considered when interpreting results from arcsine conversion.

6 ILR transformation

The Isometric Log-Ratio (ILR) transformation is an essential technique in compositional data analysis. It is designed to transform compositional vectors, which are constrained by a constant sum, into real-valued vectors that can be analyzed using standard statistical methods. The ILR transformation in matrix form is expressed as:

$$\mathrm{ILR}(X) = \mathrm{CLR}(X) \cdot H$$

In this formulation, H represents an orthonormal contrast matrix of size $p \times (p-1)$, where each column is orthogonal to the vector of ones, $\mathbf{1}_p$. A common and effective choice for H is the transposed Helmert sub-matrix. This Helmert sub-matrix is obtained by removing the first row of the Helmert matrix, providing a convenient orthonormal basis for the transformation [4, 5].

The ILR transformation facilitates the application of conventional statistical techniques to compositional data by eliminating the constant sum constraint, thus converting the data into a more analytically tractable form.

A Helmert matrix [4] M_p of order p is a $p \times p$ orthogonal matrix, commonly used in various statistical and mathematical applications, including the ILR transformation. The matrix M_p is defined as follows:

$$M_p = \begin{pmatrix} \frac{1}{\sqrt{p}} & \frac{1}{\sqrt{p}} & \frac{1}{\sqrt{p}} & \cdots & \frac{1}{\sqrt{p}} \\ -\sqrt{\frac{1}{2}} & \sqrt{\frac{1}{2}} & 0 & \cdots & 0 \\ -\sqrt{\frac{1}{6}} & -\sqrt{\frac{1}{6}} & \frac{2}{\sqrt{6}} & \cdots & 0 \\ \vdots & \vdots & \vdots & \ddots & \vdots \\ -\sqrt{\frac{1}{(p-1)p}} & -\sqrt{\frac{1}{(p-1)p}} & -\sqrt{\frac{1}{(p-1)p}} & \cdots & \sqrt{\frac{p-1}{p}} \end{pmatrix}_{p \times p},$$

And the transposed Helmert sub-matrix H is defined as:

$$H = \begin{pmatrix} -\sqrt{\frac{1}{2}} & -\sqrt{\frac{1}{6}} & \cdots & -\sqrt{\frac{1}{(p-1)p}} \\ \sqrt{\frac{1}{2}} & -\sqrt{\frac{1}{6}} & \cdots & -\sqrt{\frac{1}{(p-1)p}} \\ 0 & \sqrt{\frac{2}{6}} & \cdots & -\sqrt{\frac{1}{(p-1)p}} \\ \vdots & \vdots & \ddots & \vdots \\ 0 & 0 & \cdots & \sqrt{\frac{p-1}{p}} \end{pmatrix}_{p \times (p-1)},$$

If the full transposed Helmert matrix were used after the CLR transformation, one of the resulting columns would be entirely zeros because the CLR already enforces the sum of zero constraint. To avoid this redundancy, we use the transposed Helmert sub-matrix H, which excludes the all-zero column and provides an appropriate orthonormal basis for the ILR transformation.

Given the compositional vector $X = (x_1, x_2, \ldots, x_p)$, the Centered Log-Ratio (CLR) transformation is:

$$\mathrm{CLR}(X) = \left(\log\left(\frac{x_1}{g(X)}\right), \log\left(\frac{x_2}{g(X)}\right), \ldots, \log\left(\frac{x_p}{g(X)}\right)\right),$$

where $g(X)$ is the geometric mean of X:

$$g(X) = \left(\prod_{i=1}^{p} x_i\right)^{1/p}.$$

The first column of the transposed Helmert matrix M_p^T is:

$$\left(\frac{1}{\sqrt{p}}, \frac{1}{\sqrt{p}}, \ldots, \frac{1}{\sqrt{p}}\right)^T.$$

Now, the product of the CLR-transformed vector with this first column is:

$$\mathrm{CLR}(X) \cdot \left(\frac{1}{\sqrt{p}}, \frac{1}{\sqrt{p}}, \ldots, \frac{1}{\sqrt{p}}\right)^T = \sum_{i=1}^{p} \left(\log\left(\frac{x_i}{g(X)}\right) \cdot \frac{1}{\sqrt{p}}\right).$$

Since the sum of the CLR components is zero:

$$\sum_{i=1}^{p} \log\left(\frac{x_i}{g(X)}\right) = 0,$$

the product becomes:

$$\sum_{i=1}^{p}\left(\log\left(\frac{x_i}{g(X)}\right)\cdot\frac{1}{\sqrt{p}}\right)=0.$$

This shows that the result of the multiplication is zero, demonstrating why the first column of M_p^T is excluded in the ILR transformation.

The Helmert matrix is specifically structured to maintain orthogonality, making it a powerful tool for transforming and analyzing data. Each element of the matrix is constructed to ensure that the rows are orthogonal to each other, facilitating efficient and meaningful data transformations.

7 Algorithm for Zero Inflated Negative Binomial Regression Simulation

To simulate data using a zero-inflated negative binomial distribution model, we use the following parameters:

- n: total number of observations
- p: number of variables
- β_0: intercept for generating means
- β: coefficient for significant variables
- α: dispersion parameter
- q: probability of zero inflation

The detailed algorithm provides a step-by-step guide to simulate zero-inflated negative binomial data, ensuring clarity and ease of replication.

Algorithm 3 Simulate Zero-Inflated Negative Binomial Data

Input: $n, p, \beta_0, \beta, \alpha, q$
Initialize: Data frame to store results
Split n into two groups: $n1 = n/2$, $n2 = n/2$
Create a covariate vector x with $n1$ ones and $n2$ zeros
Generate Significant Group:
Compute means $\mu_{\text{Significant}} = \exp(\beta_0 + x \cdot \beta)$
Compute size parameter $\text{size}_{\text{Significant}} = 1/\alpha$
Compute probability parameter $\text{prob}_{\text{Significant}} = 1/(\alpha \cdot \mu_{\text{Significant}} + 1)$
Generate $p/2$ non-zero variables using negative binomial distribution xn
Generate Insignificant Group:
Compute means $\mu_{\text{Insignificant}} = \exp(\beta_0 + x \cdot 0)$
Compute size parameter $\text{size}_{\text{Insignificant}} = 1/\alpha$
Compute probability parameter $\text{prob}_{\text{Insignificant}} = 1/(\alpha \cdot \mu_{\text{Insignificant}} + 1)$
Generate $p/2$ Insignificant variables using negative binomial distribution xz
Combine significant and insignificant variables into a matrix X
Apply Zero-Inflation:
Generate a matrix π of zero proportions with probability q by Bernoulli distribution
Multiply each column of X by its corresponding column in π to obtain Xc
Convert Data:
Generate scaling factors s from a log-normal distribution
Scale Xc by s
Convert rows of Xc to obtain Xp
Output: Simulated data frame with zero-inflated negative binomial data

8 False Discovery Rate Analysis of Compositional Data Transformations In Zero Inflated Negative Binomial Regression Simulation

Table S3: Coefficients from Linear Regression Models for False Discovery Rate (FDR) Using Various Compositional Data Transformations

Transformation	Intercept	α	β_0	β	q
ALR	0.1021223188	0.0049757738	0.0105717212	-0.0260431012	0.1390237909
CLR	0.4490747879	0.0118070074	-0.0137190817	0.0275921514	0.3659374859
Additive Logit Contrast	0.0950694869	0.005418065	0.009872983	-0.0247924912	0.1374943159
Centered Logit Contrast	0.4467507697	0.0122274497	-0.0128777303	0.0267945591	0.3767033653
Additive Arcsine Contrast	0.0922344385	-8.26897E-05	-0.0006005535	-0.0090868297	0.021137927
Centered Arcsine Contrast	0.5631169016	-0.0001472267	0.0065697908	0.0319505568	-0.0692192887
Additive Power Contrast	0.1532115708	0.01895056	0.0022263147	-0.0377870763	0.5360578439
Centered Power Contrast	0.3259994827	0.0140623529	-0.0184409646	0.0221695655	0.355284596
Additive Dual Group Logit Contrast	0.1258790405	0.00314871	0.0013079828	-0.0195125129	0.0906221926
Centered Dual Group Logit Contrast	0.6001761009	-0.0042382668	0.0043775963	0.018904747	-0.2600715616
Additive Dual Group Power Contrast	0.1287946116	0.0065918668	-0.0122038661	0.0130605316	0.4365981702
Centered Dual Group Power Contrast	0.438276361	-0.004221537	-0.0135771141	0.0120599281	0.1120564707
ILR	0.8577421634	-0.0045186191	-0.0085389122	0.0276596558	-0.1683786731
Boxcox in Ratio	0.1043454477	0.0051344852	0.0095014293	-0.0264597341	0.1640017802
Dual Group Boxcox in Ratio	0.0949341647	0.004797661	0.0106761878	-0.0253652202	0.1567362638

The table of coefficients from linear regression models for False Discovery Rate (FDR) using various compositional data transformations reveals several key insights. The Additive Arcsine Contrast has the lowest FDR baseline and is least sensitive to α (precision) and q (percentage of zeros), indicating it is a reliable transformation method for compositional data. Additionally, the Additive Logit Contrast shows a relatively low baseline in FDR, which is favorable; however, it has a high sensitivity to q, with a value of 0.1374943159, meaning it is highly sensitive to the presence of zeros.

Centered Contrast Transformations, such as the Centered Log-Ratio (CLR), are generally more robust and less sensitive to outliers. However, when variables in one group consistently show higher or lower counts compared to another group, Additive Contrast transformations, like the Additive Log-Ratio (ALR), become more favorable over CLR. This is because CLR transformation averages all variables, potentially diluting the effect of truly significant changes and amplifying noise.

Furthermore, the Isometric Log-Ratio (ILR) transformation is not well-suited for differential abundance testing in compositional data, as evidenced by its less favorable performance metrics in the table.

9 Comparison of Compositional Data Transformations Using GUniFrac in False Discovery Rate Analysis

Table S4: Coefficients from Analysis of False Discovery Rate (FDR) Using Various Compositional Data Transformations with GUniFrac. The table presents the intercept and coefficients for each transformation method, with parameters including Differential OTU Direction (unbalanced), Differential OTU Mode (abundant, mix, rare), Average Sequencing Depth, Sequencing Depth Dispersion, Covariate Effect Variability, Confounder Effect Variability, and Sequencing Depth-Covariate Dependence.

Transformation	Intercept	Differential OTU Direction (unbalanced)	Differential OTU Mode (mix)	Differential OTU Mode (rare)	Average Sequencing Depth	Sequencing Depth Dispersion	Covariate Effect Variability	Confounder Effect Variability	Sequencing Depth-Covariate Dependence
ALR	0.0501573	0.0325691	0.0907699	0.6882531	-0.0000116	-0.0005160	-0.0928291	0.1394318	0.1986776
CLR	0.1113279	0.0382828	0.0484900	0.3748910	-0.0000264	-0.0001797	-0.0734211	0.1605696	0.6773365
Additive Logit Contrast	0.0504786	0.0328908	0.0891064	0.6882027	-0.0000115	-0.0006324	-0.0896854	0.1428289	0.1959956
Centered Logit Contrast	0.1122209	0.0396678	0.0472973	0.3747043	-0.0000264	-0.0002848	-0.0715049	0.1635313	0.6658891
Additive Arcsine Contrast	0.0225901	0.1057702	0.0268758	0.6728866	-0.0000053	-0.0013283	-0.1051854	0.2077370	0.0079953
Centered Arcsine Contrast	0.1160494	0.0201841	0.0150918	0.3750728	-0.0000244	-0.0005435	-0.0722162	0.2394557	0.2414411
Additive Power Contrast	0.4022393	0.0913211	-0.0576783	0.0793490	0.0000154	0.0037218	-0.0634193	0.0532902	0.7329875
Centered Power Contrast	0.3448103	0.0309109	-0.1321974	-0.0093925	0.0000165	0.0034716	-0.0600803	0.0654061	1.0194538
Additive Dual Group Truncated Logit Contrast	0.2167946	0.2648236	0.1290733	0.5796903	0.0000014	0.0010184	-0.1352356	0.0738215	-0.0441590
Centered Dual Group Truncated Logit Contrast	0.1741270	0.0808205	0.0727550	0.5274257	-0.0000064	0.0020916	-0.1246561	0.1351361	0.0055332
Additive Dual Group Box-Cox Contrast	0.7555022	0.0409592	0.0196795	0.0180375	0.0000037	0.0053057	0.0021546	0.0255372	0.0824044
Centered Dual Group Box-Cox Contrast	0.7663530	-0.0091691	0.0248464	-0.0112267	0.0000027	0.0045974	-0.0004710	0.0285720	0.1193889
ILR	0.7025177	0.0086573	0.1678572	0.1178134	-0.0000046	0.0004400	-0.0160063	0.0180228	0.1158913
Box-Cox in Ratio	0.0306066	0.0498842	0.0941011	0.7136711	-0.0000092	-0.0009641	-0.0807167	0.1382313	0.1656883
Dual Group Box-Cox in Ratio	0.0576113	0.0343120	0.0890256	0.6729843	-0.0000110	-0.0014138	-0.0897438	0.1452335	0.1551161

The table presents the coefficients from a linear regression analysis of False Discovery Rate (FDR) using various compositional data transformations with GUniFrac. Several key observations can be drawn from this analysis. The Additive Contrast framework generally exhibits lower FDR compared to the Centered Contrast framework. This difference can be attributed to the inherent averaging mechanism of the Centered Contrast, which tends to dilute the effects of truly significant changes and amplify noise. This averaging can obscure true signals, leading to higher FDR. Among the various transformations, the Additive Arcsine Contrast demonstrates the lowest FDR. This suggests that for researchers prioritizing the minimization of false discoveries, the Additive Arcsine Contrast is the most suitable choice. Conversely, the Centered Arcsine Contrast, while having a slightly higher FDR, may offer higher statistical power. This makes it a preferable option when the objective is to maximize the detection of true signals despite a marginal increase in FDR.

10 Distribution Characteristics of Transformed Human Gut Microbiota Data in IBD Studies [6]

Table S5: Mean and standard deviation (SD) of skewness and kurtosis for different transformation methods applied to real microbiome data. The table includes values for two groups (A and B), highlighting the distribution characteristics of the transformed data.

Transformation	Group	Mean_Skewness	SD_Skewness	Mean_Kurtosis	SD_Kurtosis
Original	A	2.50054191599006	0.948832163410656	8.96215509002084	4.49240421223716
	B	0.300135384674107	1.18768446472988	4.28925602974769	2.78543399581582
ALR	A	0.958279077166417	0.984633586455365	3.81728543570505	2.58035049792459
	B	0.30668007526582	1.1898941565579	4.30221437350457	2.78950189649782
CLR	A	0.671719273515621	1.41608399306025	5.42109098068486	3.16222893641763
	B	0.966354910928452	0.980950048838877	3.83233703311417	2.58964243482295
Additive Logit Contrast	A	1.41074421398215	0.875556154500528	4.85579177844521	3.22711144287483
	B	-1.06402721379327	0.866139031664807	5.27475973769142	1.24722300547628
Centered Logit Contrast	A	0.282670391007251	1.11332734230947	4.18445466500169	2.67298457314265
	B	1.70598353587872	1.55782175904548	7.08733315971486	4.74370349945543
Additive Arcsine Contrast	A	0.231318635324607	1.46058782328885	4.89815787527713	2.06113832711452
	B	0.266425307354239	1.11327820614344	4.18963873450135	2.67445014430139
Centered Arcsine Contrast	A	1.45584426115118	1.03522816245309	5.30749216530662	3.69875070039674
	B	0.965683266868988	1.02502187361898	3.92908582957126	2.78323976410813
Additive Power Contrast	A	2.50647712804456	1.18828210472651	12.2174298624603	9.96318292402064
	B	1.72113586438852	0.871870349729693	6.62190441667437	4.00078107461579
Centered Power Contrast	A	-0.406292013021907	0.197556521677653	3.72551197001668	1.47220062342001
	B	0.638226013540565	0.865068575706735	4.13485227634258	1.3758303110139
Additive Dual Group Logit Contrast	A	3.45975945015708	1.8616431286431	22.0596450523156	22.340758847754
	B	-0.407045002164373	0.200515590490373	3.73334472514277	1.50708792642707
Centered Dual Group Logit Contrast	A	0.649806173385068	0.781134293093321	3.93917669218685	1.16886116661707
	B	-0.164985191373388	0.511297160328494	7.73595551375995	3.15710750489974
Additive Dual Group Power Contrast	A	-0.529634312112227	0.457185834547291	3.18429820811398	0.842334707226022
	B	0.818146743505249	0.578922972810268	3.73006352465017	1.59111805420648
Centered Dual Group Power Contrast	A	6.49368393297039	2.63826839674181	57.5622062922592	42.1847310075351
	B	0.0167517113431521	0.824575245109452	7.49655385869227	3.487481816403
ILR	A	-0.0542654073803328	0.177187635772774	7.36310601639588	2.87805007694122
	B	0.156852384593474	1.58331127007885	12.0093598931527	7.08517180339674
Boxcox in Ratio	A	1.76403094940378	0.820356181416003	6.68648462976225	4.04357271991319
	B	0.0279411904912588	0.838571367454622	7.56385166561939	3.51415821716388
Dual Group Boxcox in Ratio	A	0.744006440968648	2.63830165600976	20.1015007047139	14.6315898576981
	B	1.78411659663587	0.823140217742411	6.80679682399804	4.16776557599094

From the table, we can observe that the normalization power for ALR, CLR, Additive Logit Contrast, Centered Logit Contrast, Additive Arcsine Contrast, and Centered Arcsine Contrast is very close, and they all perform well. One key aspect to notice is that, besides normalization power, it is essential to ensure that the signal remains intact after transformation. As shown in our main analysis, the Centered Arcsine Contrast demonstrates the highest overlap with the DESeq2-detected significant results in both groups while maintaining a relatively low number of false positives detected only by the t-test. This indicates that the Centered Arcsine Contrast not only normalizes the data effectively but also preserves the biological signal, making it a robust choice for further statistical analysis.

11 Impact of Zero Replacement in ALR and CLR Transformations

In our study, we examined the impact of zero replacement on Additive Log Ratio (ALR) and Centered Log Ratio (CLR) transformations concerning the power and false discovery rate (FDR) of differential abundance testing. To this end, we conducted a simulation study where we generated synthetic microbiome data with varying proportions of zero-inflation (q) and applied different constants for zero replacement.

The data were generated using a zero-inflated negative binomial (ZINB) model with a sample size of 100 and 50 variables, of which 25 were designated as significant. We replaced zeros in the data with a constant and applied ALR and CLR transformations. For each transformed dataset, we performed t-tests to evaluate differential abundance, calculating power and FDR for each scenario. This process was repeated for 300 simulations across different values of q (0, 0.3, 0.5, 0.7) and constants (0.1, 0.5, 1, 2, 5). For a detailed algorithm on ZINB simulation, refer to Supplementary Material Section 4.

The results from these simulations were analyzed using ANOVA to quantify the effects of zero-inflation and the zero-replacement constant on power and FDR. The models included terms for q (proportions of zero-inflation) and the constant (values used to replace zeros in the data), and the p-values were recorded.

Method	Response	q	Constant
ALR	Power	5.74e-10	0.00123
ALR	FDR	1.39e-06	0.0227
CLR	Power	1.99e-12	0.0143
CLR	FDR	2.08e-09	0.0041

Table S6: Impact of Zero Replacement on Power and FDR in ALR and CLR Transformations

The Table S6 summarizes the findings from these models. The significant impact of the zero-replacement constant on both power and FDR in ALR and CLR transformations underscores the challenges associated with zero handling in compositional data analysis. These results emphasize the need for careful consideration and appropriate methodological adjustments to avoid false discoveries and ensure accurate detection of differentials in microbiome studies.

12 Formulas for Proposed Transformations

1. Additive Contrast Transformations

For compositional data X, the additive contrast matrix is defined as:

$$C = \begin{pmatrix} 1 & 0 & 0 & \cdots & 0 & 0 \\ 0 & 1 & 0 & \cdots & 0 & 0 \\ 0 & 0 & 1 & \cdots & 0 & 0 \\ \vdots & \vdots & \vdots & \ddots & \vdots & \vdots \\ 0 & 0 & 0 & \cdots & 1 & 0 \\ -1 & -1 & -1 & \cdots & -1 & 0 \end{pmatrix}_{p \times p}, \tag{1}$$

where $I_{p \times p}$ is a p-dimensional diagonal matrix, and the last row sums to zero.

Transformation Types

- **Additive Power Contrast (APC)**: Power$(X)c_j$
- **Additive Logit Contrast (ALTC)**: $logit(X)c_j$
- **Additive Arcsine Contrast (AAC)**: $arcsine(X)c_j$
- **Additive Dual Group Truncated Logit Contrast (ADGTLC)**: Dual Group Truncated Logit$(X)c_j$
- **Additive Dual Group Box-Cox Contrast (ADGBCC)**: Dual Group Box-Cox$(X)c_j$

2. Centered Contrast Transformations

For compositional data X, the centered contrast matrix is defined as:

$$C = \begin{pmatrix} 1 - \frac{1}{p} & -\frac{1}{p} & -\frac{1}{p} & \cdots & -\frac{1}{p} \\ -\frac{1}{p} & 1 - \frac{1}{p} & -\frac{1}{p} & \cdots & -\frac{1}{p} \\ -\frac{1}{p} & -\frac{1}{p} & 1 - \frac{1}{p} & \cdots & -\frac{1}{p} \\ \vdots & \vdots & \vdots & \ddots & \vdots \\ -\frac{1}{p} & -\frac{1}{p} & -\frac{1}{p} & \cdots & 1 - \frac{1}{p} \end{pmatrix}_{p \times p}. \tag{2}$$

Transformation Types

- **Centered Power Contrast (CPC)**: Power$(X)c_j$
- **Centered Logit Contrast (CLTC)**: $logit(X)c_j$
- **Centered Arcsine Contrast (CAC)**: $arcsine(X)c_j$
- **Contrast Dual Group Truncated Logit Contrast (CDGTLC)**: Dual Group Truncated Logit$(X)c_j$
- **Contrast Dual Group Box-Cox Contrast (CDGBCC)**: Dual Group Box-Cox$(X)c_j$

13 Potential Usefulness of the New Framework in Distance-Based Methods

In this supplementary section, we want to show how our new framework can be used in distance-based approaches, such as Euclidean distances. For this comparison, we take the traditional method CLR and our new proposed method CAC as examples. We use the Hadza microbiome dataset for this demonstration, which is sourced from Smits et al. [7], Zhang et al. [8]. The dataset is from Hadza hunter-gatherers of Tanzania, collected across five seasonal groups: 2013-LD (Late dry), 2014-EW (Early wet), 2014-LW (Late wet), 2014-ED (Early dry), and 2014-LD (Late dry) [7]. To enable a two group comparison and introduce overlapping effects, we combined the 2014 data into two categories: dry and wet seasons.

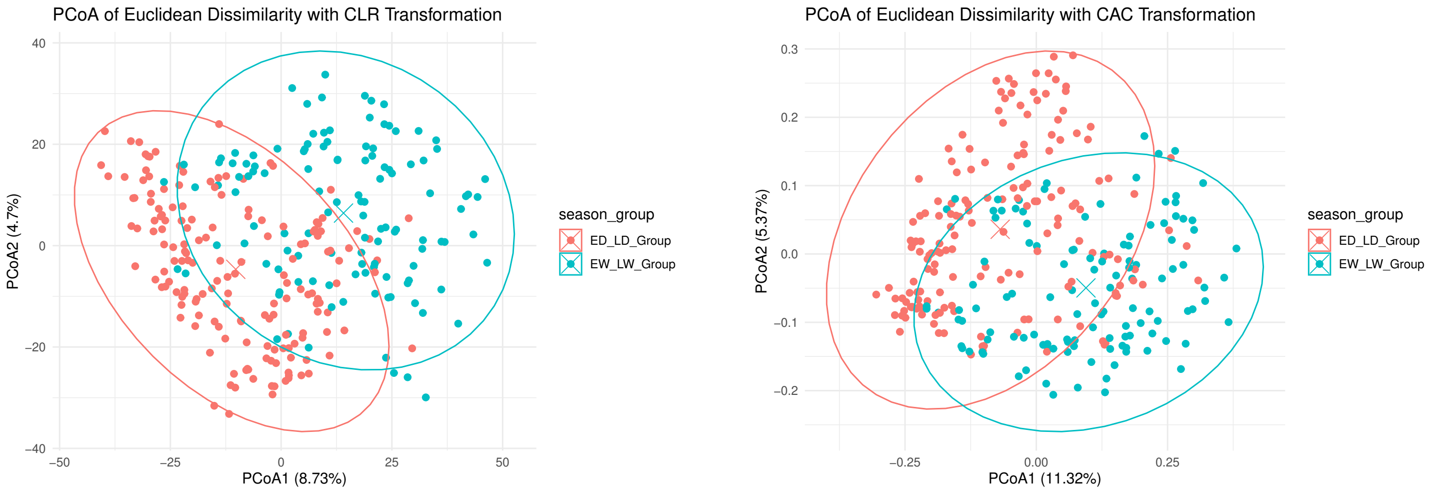

(a) PCoA Plot Using CLR Transformation

(b) PCoA Plot Using CAC Transformation

Figure S2: Comparison of PCoA plots using CLR and CAC transformations with Euclidean distance.

Figure S2a presents a Principal Coordinates Analysis (PCoA) plot based on Euclidean distances calculated from CLR-transformed microbiome data. Zero counts in the dataset were replaced with 0.5 before obtaining relative abundance and performing the transformation. Figure S2b shows the PCoA plot based on Euclidean distances calculated from CAC-transformed microbiome data. It represents the relationships between samples under the CAC transformation.

A key difference between CLR and CAC is that CLR requires pseudo-counts, whereas CAC does not. Whether pseudo-count values significantly influence distance calculations is also a key concern. Following the visualization in Figures S2a and S2b, we conducted an analysis to investigate the effect of different pseudo-count values on microbiome data transformed using the CLR method. For this analysis, we used pseudo-count values of 0.1, 0.3, 0.5, 0.7, 0.9, 1.1, 1.3, 1.5, 1.7, and 1.9. We replaced zeros in the count data with each of these pseudo-count values and then applied the CLR transformation. After each transformation, the Euclidean distance between samples was calculated to assess the dissimilarity introduced by varying pseudo-counts.

To evaluate whether the variability in Euclidean distances was significantly influenced by the choice of zero-replacement value, we performed a PERMANOVA (Permutational Multivariate Analysis of Variance) using the `adonis2` function in R. In this test, the zero replacement value served as the predictor variable, and the distance matrix as the response variable. This analysis allowed us to assess if differences in Euclidean distances between samples were significantly affected by the choice of pseudo-count, potentially impacting the overall structure of the CLR-transformed data and relationships among samples.

The PERMANOVA results indicated that the zero-replacement values had a significant impact on the structure of the CLR-transformed data, with an F-value of **28.658** and a p-value of **0.001**, suggesting that the choice of pseudo-count meaningfully alters the relationships between samples.

In contrast, the CAC method does not require pseudo-counts, eliminating the potential variability caused by zero-replacement. As shown in Figure S2, the Euclidean distances computed from CAC-transformed data, which is similar to CLR-transformed data, remained stable, highlighting the robustness of our method in handling zero-inflated microbiome datasets.

14 Enhancing Variable Selection in Compositional Data: Integrating Additive Arcsine Contrast (AAC) into the Linear Log-Contrast Model

The linear log-contrast model [9, 10] is a regression framework designed for compositional data, where the covariates are proportions that sum to one. Traditional regression techniques fail to handle the inherent dependencies among these components, but the linear log-contrast model addresses this by using log-ratio transformations [11]. The model is expressed as $y = Z\beta + \epsilon$ where Z is the matrix of log-transformed covariates, β is the vector of regression coefficients, and ϵ is the error term. The coefficients β are subject to the zero-sum constraint $\sum_{j=1}^{p} \beta_j = 0$ This constraint ensures that the model is scale-invariant, focusing on the relative proportions of the covariates rather than their absolute values.

In high-dimensional settings, variable selection is crucial to identifying the most relevant covariates. This is achieved through l_1*-regularization* (lasso) [12], which induces sparsity by forcing some coefficients to be exactly zero. The optimization problem for variable selection is formulated as [10]:

$$\hat{\beta} = \arg\min_{\beta} \left(\frac{1}{2n} \|y - Z\beta\|_2^2 + \lambda \|\beta\|_1 \right), \text{ subject to: } \sum_{j=1}^{p} \beta_j = 0.$$

Here, λ is a regularization parameter that controls the trade-off between fitting the model and promoting sparsity, ensuring that only the most important log-ratios are selected [10].

Our proposed method, the Additive Arcsine Contrast (AAC), can effectively replace the traditional Additive Log-Ratio (ALR)transformation in the linear log-contrast model, offering a significant advantage by eliminating the need for zero-replacement, a common issue in compositional data like microbiome datasets. In the ALR transformation, for a composition $X = (X_1, X_2, \ldots, X_p)$, where $\sum_{j=1}^{p} X_j = 1$, the transformed variables are typically defined as $Z_j = \log\left(\frac{X_j}{X_p}\right)$, where X_p serves as the reference component. While effective, this transformation is problematic for zeros because $\log(0)$ is undefined, necessitating complex zero-replacement strategies that can introduce bias or distort the data.

In contrast, the AAC transformation handles zeros naturally. For the same composition X, we define the AAC transformation as

$$Z_j = \frac{2}{\pi} \arcsin\left(\sqrt{X_j}\right) - \frac{2}{\pi} \arcsin\left(\sqrt{X_p}\right),$$

where $\arcsin(0) = 0$ ensures that the transformation is valid even when $X_j = 0$. By substituting this arcsine-based transformation in the linear log-contrast model, the response y becomes

$$y_i = \frac{2}{\pi} \left(\arcsin\left(\sqrt{X_{ij}}\right) - \arcsin\left(\sqrt{X_{ip}}\right) \right) \eta + \epsilon_i,$$

which maintains the original structure of the model. This preserves the relative relationships between components, similar to the log-ratio transformation, but with the advantage of handling zeros more robustly.

Both AAC and ALR transformations preserve the parameters' dimensionality, ensuring that the degrees of freedom remain unchanged. In both approaches, we have $p - 1$ transformed variables corresponding to the non-reference components. Therefore, the AAC method, like ALR, retains the same number of degrees of freedom as the original model, preserving statistical power without altering the model's complexity.

We can reparameterize the above to obtain the generalized form

$$y_i = \left(\frac{2}{\pi} \arcsin\left(\sqrt{x_{ij}}\right) \right) \beta + \epsilon_i, \text{ subject to: } \sum_{j=1}^{p} \beta_j = 0.$$

Using this regression model, we can then apply l_1*-regularization* and conduct variable selection.

In summary, replacing ALR with AAC in the linear log-contrast model is fully justified, as AAC simplifies zero handling while preserving the essential properties of the model, including its structure and degrees of freedom. The key advantage of AAC over ALR is its natural ability to handle zeros without requiring zero-replacement strategies, which can introduce bias or distort the data. This makes the AAC method particularly suitable for zero-heavy datasets like those commonly encountered in microbiome studies, allowing for efficient variable selection while maintaining the integrity of the model's structure.

15 Visualization of Transformed Microbiome Data Using Various Methods

This viaualization uses the human gut metagenome dataset, primarily focused on Inflammatory Bowel Disease (IBD), which includes Ulcerative Colitis (UC) and Crohn's Disease (CD) [6]. Initially, the dataset, comprising 206 samples, was reorganized by consolidating diagnostic groups into two categories: healthy controls and all other diagnoses. Rows with more than 90% zero values were filtered out, reducing the taxa from 7019 to 211.

We applied DESeq2, using the 'poscounts' estimator to handle zeros by calculating a modified geometric mean [13], supported by Van den Berge et al. [14]. This method identified significant taxa, such as taxa number 197 after filtering[1].

We selected this taxon to visualize the data distribution after transformation using various methods. To tackle the challenges of visualizing high-dimensional, compositional microbiome data, we focused on a single variable post-transformation to represent the data distribution effectively. The p-value in the figure represents the results of a two-sample t-test applied after each transformation method. We chose the two-sample t-test because it assumes the data follow a normal distribution, making it appropriate for comparing transformed data between the healthy controls and other diagnoses.

[1] Taxa 197 sequence: TACGTAGGTCCCGAGCGTTGTCCGGATTTATTGGGCGTAAAGCGAGCGCAGGCGGTTTGATAAGTCTGAAGTTAAAGGCTGTG-GCTCAAC-CATAGTTCGCTTTGGAAACTGTCAAACTTGAGTGCAGAAGGGGAGAGTGGAATTCCATGT

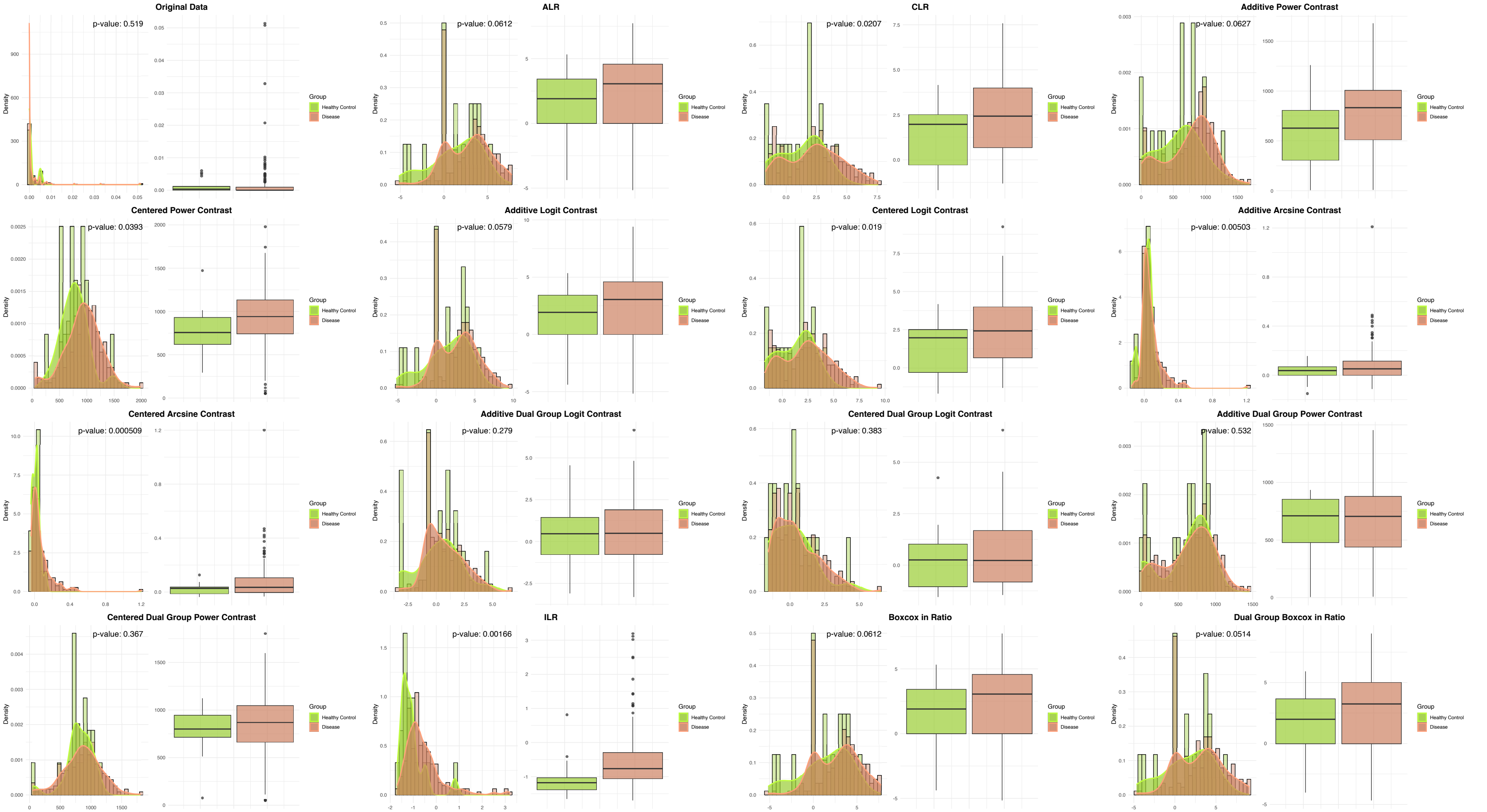

Figure S3: Combined Plots for Different Transformations

References

[1] Edwin L Crow and Kunio Shimizu. *Lognormal distributions*. Marcel Dekker New York, 1987.

[2] Jhon Atchison and Sheng M Shen. Logistic-normal distributions: Some properties and uses. *Biometrika*, 67(2):261–272, 1980.

[3] Erick Riquelme, Yu Zhang, Liangliang Zhang, Maria Montiel, Michelle Zoltan, Wenli Dong, Pompeyo Quesada, Ismet Sahin, Vidhi Chandra, Anthony San Lucas, et al. Tumor microbiome diversity and composition influence pancreatic cancer outcomes. *Cell*, 178(4):795–806, 2019.

[4] Michail T Tsagris, Simon Preston, and Andrew TA Wood. A data-based power transformation for compositional data. *arXiv preprint arXiv:1106.1451*, 2011.

[5] HO Lancaster. The helmert matrices. *The American Mathematical Monthly*, 72(1):4–12, 1965.

[6] Robert H Mills, Parambir S Dulai, Yoshiki Vázquez-Baeza, Consuelo Sauceda, Noëmie Daniel, Romana R Gerner, Lakshmi E Batachari, Mario Malfavon, Qiyun Zhu, Kelly Weldon, et al. Multi-omics analyses of the ulcerative colitis gut microbiome link bacteroides vulgatus proteases with disease severity. *Nature microbiology*, 7(2):262–276, 2022.

[7] Samuel A Smits, Jeff Leach, Erica D Sonnenburg, Carlos G Gonzalez, Joshua S Lichtman, Gregor Reid, Rob Knight, Alphaxard Manjurano, John Changalucha, Joshua E Elias, et al. Seasonal cycling in the gut microbiome of the hadza hunter-gatherers of tanzania. *Science*, 357(6353):802–806, 2017.

[8] Liangliang Zhang, Yushu Shi, Kim-Anh Do, Christine B Peterson, and Robert R Jenq. Progperm: Progressive permutation for a dynamic representation of the robustness of microbiome discoveries. *BMC bioinformatics*, 22:1–21, 2021.

[9] John Aitchison and John Bacon-Shone. Log contrast models for experiments with mixtures. *Biometrika*, 71(2):323–330, 1984.

[10] Wei Lin, Pixu Shi, Rui Feng, and Hongzhe Li. Variable selection in regression with compositional covariates. *Biometrika*, 101(4):785–797, 2014.

[11] John Aitchison. The statistical analysis of compositional data. *Journal of the Royal Statistical Society: Series B (Methodological)*, 44(2):139–160, 1982.

[12] Robert Tibshirani. Regression shrinkage and selection via the lasso. *Journal of the Royal Statistical Society Series B: Statistical Methodology*, 58(1):267–288, 1996.

[13] Simon Anders and Wolfgang Huber. Differential expression analysis for sequence count data. *Nature Precedings*, pages 1–1, 2010.

[14] Koen Van den Berge, Charlotte Soneson, Michael I Love, Mark D Robinson, and Lieven Clement. zinger: unlocking rna-seq tools for zero-inflation and single cell applications. biorxiv. *Preprint*, 10:157982, 2017.
